# Vitamin E and Fatty Acid Intake and Cardiometabolic Multimorbidity Risk: The Mediating Role of Plasma Lipid Metabolites

**DOI:** 10.3390/ijms262311477

**Published:** 2025-11-27

**Authors:** Yannan Zhang, Yangyang Cen, Xiaoxia Li, Bowen Yang, Kaijun Xing, Linxi Lian, Yongjie Yu, Yi Zhao

**Affiliations:** 1 School of Public Health, Ningxia Medical University, Yinchuan 750004, China; yannan_jn@163.com (Y.Z.);; 2 Ningxia Key Laboratory of Environmental Factors and Chronic Disease Control, Yinchuan 750004, China; 3 School of Pharmacy, Ningxia Medical University, Yinchuan 750004, China

**Keywords:** vitamins, vitamin E, fatty acids, lipid metabolomics, cardiometabolic multimorbidity, mediation analysis

## Abstract

Cardiometabolic multimorbidity (CMM), defined as the co-occurrence of two or more of the following: diabetes, ischemic heart disease, stroke, and other cardiovascular diseases, is a leading cause of global mortality. Although vitamins and fatty acid are known to influence cardiometabolic diseases through lipid metabolism, the mediating role of lipid metabolites in linking dietary vitamins and fatty acid intake to CMM remains unclear. We conducted a case–control study based on the Ningxia cohort, including 200 patients with CMM and 200 age- and sex-matched controls. Dietary intake was assessed using a semi-quantitative food frequency questionnaire, and plasma lipid profiles were analyzed using ultra-performance liquid chromatography–tandem mass spectrometry. We assessed associations between dietary nutrients and CMM using logistic regression and their relationships with lipid metabolites via Pearson correlation. Mediation analysis identified lipid metabolites that link vitamin and fatty acid intake to CMM, and these mediators were incorporated into a predictive model. We found that higher intake of vitamin E (VE), total fatty acids (FA), polyunsaturated fatty acids (PUFA), and monounsaturated fatty acids (MUFA) was significantly associated with lower CMM risk (OR < 1, FDR ≤ 0.05). Lipid profiling identified 349 differentially abundant metabolites, primarily triglycerides and diglycerides, elevated in CMM patients. Mediation analysis revealed 292 significant pathways: dietary intake VE and PUFA was linked to lower CMM risk via modulation of glycerolipid metabolites, while dietary intake FA and MUFA was linked to lower risk through regulation of glycerophospholipids. A predictive model incorporating age, sex, dietary factors, and key mediating lipids achieved good discrimination (AUC: 0.765–0.812). These findings suggest that dietary intake of VE, FA, PUFA, and MUFA is associated with reduced CMM risk through modulation of plasma lipid metabolism, with specific lipid metabolites potentially acting as key mediators.

## 1. Introduction

Multimorbidity refers to concurrent presence of two or more chronic diseases in the same individual [1]. With economic and medical advancements, the aging population is growing rapidly, which in turn has led to a rise in multimorbidity among the elderly. This trend imposes a significant economic burden on both individuals and society. Cardiometabolic multimorbidity (CMM) specifically denotes the concurrent presence of two or more cardiometabolic diseases (CMDs), such as diabetes, ischemic heart disease (IHD), stroke, and other cardiovascular diseases (CVDs) [2,3]. CMDs are a leading cause of mortality globally [4]. A prospective study revealed that the mortality risk for individuals with CMD escalates with the increasing number of comorbidities and varies over time as these conditions progress [5]. Additionally, research indicates that any combination of two or more kinds of CMDs can elevate the risk of mortality and reduce life expectancy by 12 to 15 years [2]. In-depth exploration of the pathogenesis of cardiovascular diseases and the search for effective prevention and intervention measures have become key topics in the field of medical research.

Dietary factors play an extremely important role in the pathogenesis of metabolic diseases. Vitamins have multiple physiological functions, such as antioxidation and regulating cell metabolism, and can affect multiple links related to CMD, including inflammatory response, lipid metabolism and vascular endothelial function. For instance, Vitamin E (VE) can reduce the damage of oxidative stress to vascular endothelial cells and lower the risk of atherosclerosis [6,7]; Vitamin D (VD) can regulate insulin secretion by regulating calcium influx [8]. Dietary fatty acid intake also has a profound impact on cardiovascular health. Different types of fatty acids (such as saturated fatty acids (SFA), unsaturated fatty acids (UFA), etc.) have complex and diverse metabolic processes in the human body. They not only participate in energy supply and the composition of cell membrane structure [9,10], but also affect the risk of cardiovascular diseases and diabetes by regulating blood lipid levels and other pathways [11,12]. For example, excessive intake of SFA is often closely related to dyslipidemia and atherosclerosis [13,14]. Moderate intake of UFA is not only considered to have the potential benefit of reducing the risk of cardiovascular diseases [15] but can also increase the insulin sensitivity among those people with metabolic disorders [16].

Lipid metabolism is an important link in the human metabolic process. It breaks down, transforms and transports the lipid substances ingested to various tissues and organs to meet the energy demands of the body and maintain normal physiological functions [9]. The intake of vitamins and fatty acid and their metabolic states in the body are interrelated and influence each other, jointly acting on the structures and functions related to metabolic diseases [17]. This complex interaction network enables the intake of vitamins and fatty acids to affect the pathogenesis of cardiovascular diseases through the mediating pathway of lipid metabolism, which has become a scientific issue worthy of in-depth study.

In recent years, with the continuous development and integration of multi-disciplinary research methods such as nutritional epidemiology and metabolomics [18,19], it has provided strong technical support and research ideas for the comprehensive investigation of the relationships between vitamins and fatty acid intake, lipid metabolism and the pathogenesis of CMM. Through multi-dimensional studies such as dietary surveys of large-scale populations and blood biomarker detection, it is expected to reveal the intrinsic connections and potential mechanisms therein, providing new targets and strategies for the prevention and treatment of CMM.

Therefore, based on the above research background, we aimed to deeply explore how the intake of dietary vitamins and fatty acids is related to CMM risk through alterations in lipid metabolism. It has significant scientific significance and clinical application value for improving the theoretical system of the pathogenesis of CMM, enhancing the current situation of prevention and control of CMM, and improving people’s quality of life.

## 2. Results

### 2.1. Baseline Characteristics of the Study Population

As shown in Table 1, the majority of both the case group and the control group were elderly, with median ages of 66 years and 65 years, respectively. The general demographic characteristics, including sex, ethnicity, marital status, smoking, and physical activity, were evenly distributed between the two groups (*p* > 0.05). However, alcohol consumption differed significantly between the groups (*p* < 0.05). Notably, body mass index (BMI) was significantly higher in the case group than in the control group (*p* < 0.05). In addition, the levels of systolic blood pressure (SBP), diastolic blood pressure (DBP), fasting blood glucose (FBG), and triglycerides (TG) were also significantly higher in the case group than in the control group (*p* < 0.05), whereas high density lipoprotein-cholesterol (HDL-C) was significantly lower in the case group (*p* < 0.05). Among CMM patients, the most prevalent comorbidity was hypertension (91%), followed by diabetes (65%). The co-occurrence of diabetes and hypertension was observed in 59% of cases. Other common comorbidities included stroke, acute myocardial infarction, angina, and other CVDs.

### 2.2. Characteristics of the Dietary Vitamins and Fatty Acid Intake

As shown in Table 2, the case group exhibited significantly lower intakes of total energy, vitamin B1, niacin, folic acid, vitamin C (VC), VE, total fatty acids (FA), SFA and monounsaturated fatty acids (MUFA), compared to the control group (*p* ≤ 0.05).

### 2.3. Associations of Dietary Vitamins and Fatty Acid Intake and CMM

Logistic regression models adjusted for different covariates were used to examine the relationships between dietary vitamins and fatty acid intake and risk of CMM (Table 3). In Model 1 (crude model), the intake of VE, FA, PUFA, MUFA, and SFA was associated with a lower risk of CMM, with odds ratios (ORs) and FDRs as follows: VE (OR, 0.75 [95% CI, 0.61–0.90], FDR 0.032); FA (OR, 0.78 [95% CI, 0.65–0.94], FDR 0.038); PUFA (OR, 0.80 [95% CI, 0.66–0.96], FDR 0.045); MUFA (OR, 0.78 [95% CI, 0.65–0.94], FDR 0.038); SFA (OR, 0.80 [95% CI, 0.66–0.96], FDR 0.045). In model 2, after adjusting for sex, age and physical activity, the intake of VE, FA, PUFA, and MUFA remained significantly associated with CMM risk, while SFA intake was no longer significant: VE (OR, 0.74 [95% CI, 0.61–0.89], FDR 0.027); FA (OR, 0.78 [95% CI, 0.64–0.94], FDR 0.041); PUFA (OR, 0.79 [95% CI, 0.66–0.95], FDR 0.045); MUFA (OR, 0.78 [95% CI, 0.65–0.94], FDR 0.041). In Model 3, after further adjusting for education, nation, marital status, smoking status and alcohol consumption based on model 2, the associations between the intake of VE, FA, PUFA, and MUFA and risk of CMM remained statistically significant (FDR < 0.05).

### 2.4. Lipid Metabolomics Analysis Results

**Figure 1:**
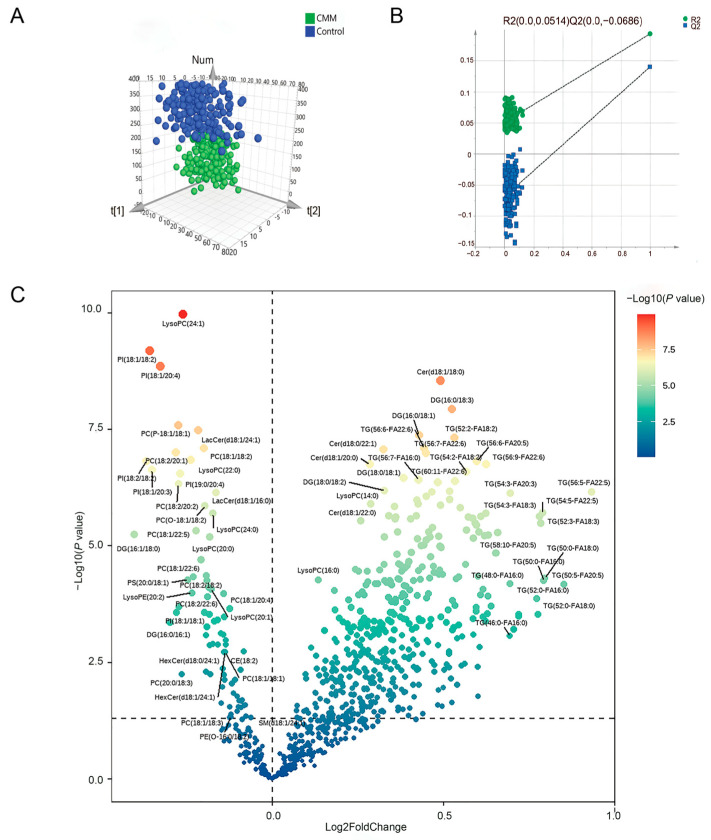
Plasma differential metabolites in patients with CMM. (**A**) Orthogonal partial least squares discriminant analysis (OPLS-DA) based on the metabolic features of plasma samples. (**B**) The performance of the OPLS-DA model. The x-axis represents the predictive ability of the model, and the y-axis represents the explanatory ability of the model. The green circles indicate the R^2^ value, which measures the explanatory power of the model. The blue squares represent the Q^2^ value, which is used to measure the predictive ability of the model. (**C**) Volcano plot. Log2FoldChange is plotted on the x-axis, where Log2FoldChange > 0 indicates higher expression levels in the disease group compared to the control group, and Log2FoldChange < 0 indicates lower expression levels in the disease group. The y-axis represents −log10(*P* value), which reflects the statistical significance of differences between the two groups; a larger −log10(*P* value) indicates a more significant difference. The magnitude of the difference is visually represented by a color gradient from blue (less significant) to red (more significant).

A total of 817 lipid metabolites were detected by non-targeted plasma metabolomics analysis. The orthogonal partial least squares discriminant analysis (OPLS-DA) pattern recognition model effectively distinguished between groups, as evidenced by the clear separation in Figure 1A, and permutation testing confirmed the model’s robustness and reliability, with no signs of overfitting (Figure 1B). Based on criteria of *p* ≤ 0.05 and variable importance in projection (VIP) > 1, a substantial number of 349 plasma lipid metabolites exhibited significant differences (Supplement Table S1). The differential metabolites between the two groups were visualized through a volcano plot (Figure 1C). Among these differential metabolites, glycerolipids constituted the largest proportion (80.2%), primarily consisting of TG and diglycerides (DG), while glycerophospholipids accounted for 17.8%, sphingolipids for 1.7%, and sterol lipids for 0.3%. As shown in the volcano plot (see Figure 1C and Supplement Table S1), the levels of glycerolipids were higher in CMM patients.

### 2.5. Associations of Dietary Vitamin and Fatty Acid Intake and Metabolites

**Figure 2:**
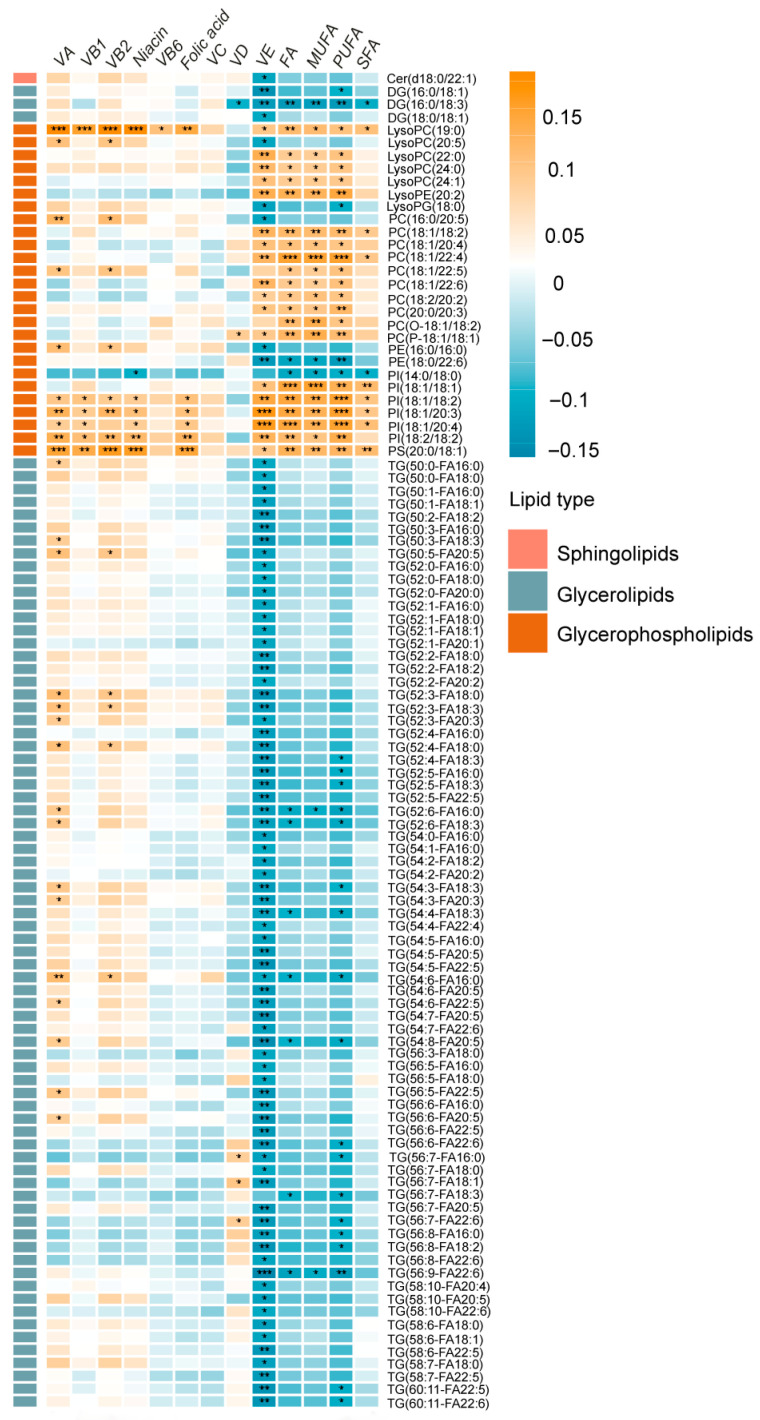
Associations of 102 differential metabolites with dietary vitamin and fatty acid intake. The color indicates the direction of the association (orange-positive and blue-negative) and the intensity (the darker the color, the stronger the association); asterisks represent the significance of the association (* *p* < 0.05, ** *p* < 0.01, *** *p* < 0.001); Metabolites are grouped by lipid class, with distinct colors representing different categories: pink for sphingolipids, blue-gray for glycerolipids, and orange-brown for glycerophospholipids.

The Pearson correlation coefficient was employed to investigate the relationships between dietary vitamins and fatty acid intake and plasma lipid metabolites, yielding a total of 273 significant associations (Figure 2). According to the results, the intake of Vitamin A (VA), VD and VE was mostly associated with plasma glycerolipids, especially DG and TG. Specifically, consumption of VA and VD showed positive associations with plasma glycerolipids, except for VD intake, which showed a negative association with DG (16:0/18:3). In contrast, VE intake exhibited a negative correlation with plasma glycerolipids. The intake of VB1, Vitamin B2 (VB2), Niacin, Vitamin B6 (VB6), and folic acid showed strong positive correlations with plasma glycerophospholipids, including lysophosphatidylcholine (LysoPC), phosphatidylinositol (PI), phosphatidylserine (PS), and phosphatidylethanolamine (PE). Notably, niacin intake and plasma PI (14:0/18:0) showed a negative correlation. Additionally, although most B vitamins were positively associated with plasma glycerolipids, the majority of these associations were not statistically significant. The consumption of FA, MUFA, and PUFA was positively associated with plasma glycerophospholipids, with the exception of Lysophosphatidylglycerol (LysoPG) (18:0), PE (18:0/22:6), and PI (14:0/18:0), which exhibited negative correlations.

### 2.6. Metabolites as a Mediator Between Dietary Vitamin and Fatty Acid Intake and CMM

**Figure 3:**
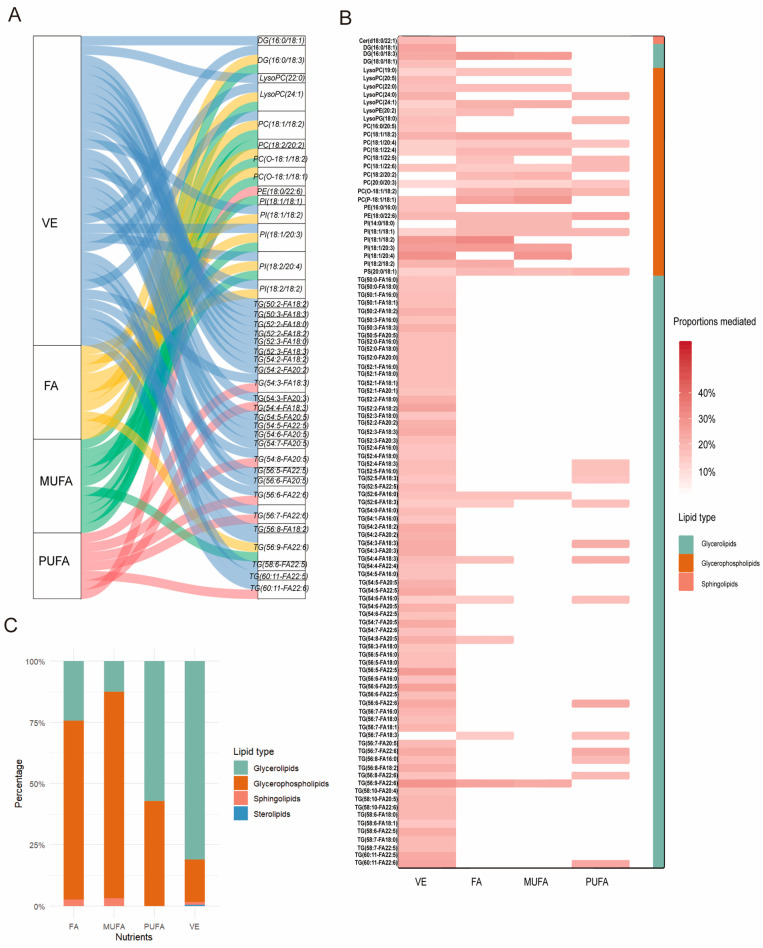
Mediation analysis identified linkages between the metabolites and dietary vitamin and fatty acid intake. (**A**) Sankey plot showing metabolite-mediated associations between dietary vitamins and fatty acid intake and CMM risk. Mediation linkages were shown with *p* total effect < 0.05, *p* mediation < 0.05 and mediated proportion > 20%. Colors corresponded to the mediation linkages mediated by different dietary vitamins and fatty acids (blue for VE, yellow for FA, green for MUFA, and red for PUFA). (**B**) Proportions of lipid metabolite classes among significant mediators linking dietary vitamin and fatty acid intake and CMM risk. Different colors represent different lipid metabolite classes. (**C**) The proportion of different lipid metabolites in the mediation of dietary vitamin and fatty acid intake on CMM risk. The mediating proportion of lipid metabolites is indicated by red shading, with darker hues signifying a stronger mediating effect.

We conducted a mediation analysis to explore whether plasma metabolites might account for part of the observed associations between dietary vitamin E and fatty acid intake and CMM risk. A total of 292 potential mediation links were identified (total effect < 0.05, mediated *p*-value < 0.05; Supplement Table S2). Among these, a total of 195 mediation links were associated with VE intake (Figure 3B), with 81% of the metabolites being glycerolipids. The highest mediation proportions were observed for PI (18:1–20:4) at 29.4% and TG (56:9-FA22:6) at 28.5%. Meanwhile, 37 mediation links were associated with FA intake, with glycerophospholipids accounting for 73% of the mediating metabolites (Figure 3B). Specifically, PI (18:1–18:2) and DG (16:0/18:3) exhibited high mediation proportions of 32% and 28%, respectively. A total of 32 pathways were related to MUFA intake, with glycerophospholipid metabolites comprising 84.3% of the mediators (Figure 3B). Notably, PI (18:1–20:4) and PC (P-18:1/18:1) had mediation proportions of 28.7% and 27.6%, respectively. A total of 28 pathways were associated with PUFA intake, with 57.1% of the mediator metabolites being glycerolipids (Figure 3B). Among these, PE (18:0/22:6) had a mediation proportion of 24%, followed by TG (60:11-FA22:6) at 23.5%. We also found that the metabolites LysPCO (22:0), PC (18:1/20:4), PC (18:1/22:6), PC (20:0/20:3), PE (18:0/22:6), PI (18:1/18:1), and PS (20:0/18:1) may collectively act as mediators of the associations between VE, FA, MUFA, and PUFA intake and CMM incidence (Figure 3C).

### 2.7. Using a Logistic Regression Model to Identify the Metabolic Characteristics of CMM

**Figure 4:**
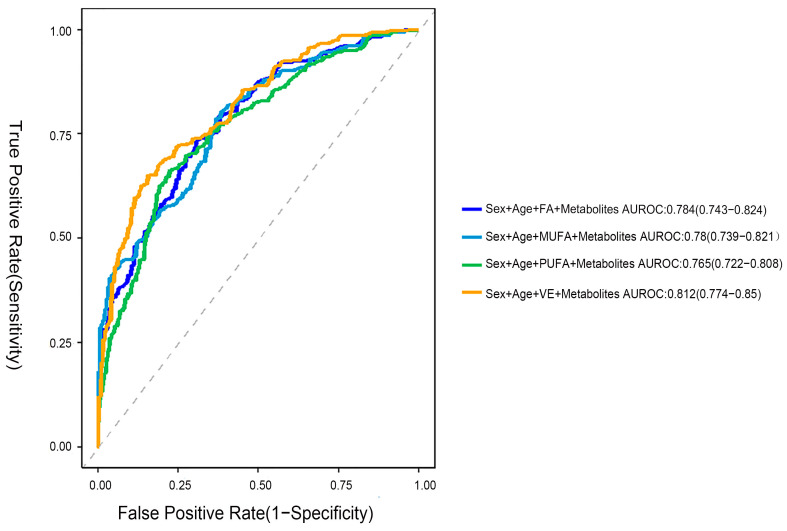
Prediction model for CMM. ROC curves comparing models that include sex, age, metabolites, and different dietary components, with each model shown in a different color.

To assess the predictive performance of dietary vitamins and fatty acid intake combined with intermediate metabolites for CMM risk, we calculated the Area Under the Receiver Operating Characteristic Curve (AUROC) for various models based on the mediation analysis results. These analyses aimed to quantify the impact of integrating multiple biomarkers on predicting CMM risk. As shown in Figure 4, the AUROC values were as follows: sex + age + FA + intermediate metabolites, 0.784 (95% CI 0.743–0.824); sex + age + MUFA + metabolites, 0.780 (95% CI 0.739–0.821); and sex+ age + PUFA + metabolites, 0.765 (95% CI 0.722–0.808). Due to the large number of intermediate metabolites involved with VE, we selected the intermediate metabolites with a fold change (FC) ≥ 1.38 for inclusion in the prediction model. The results showed that the AUROC for the model combining sex, age, VE, and intermediate metabolites (FC ≥ 1.38) reached 0.812 (95% CI, 0.774–0.850), indicating strong predictive ability.

## 3. Discussion

In this study, we explored whether plasma lipid metabolites might help explain the observed associations between dietary vitamin and fatty acid intake and CMM prevalence. We examined the dietary vitamin and fatty acid intake and plasma lipid metabolic profiles of CMM patients and identified that lipid metabolites suggest a potential mediating role in the relationship between the consumption of VE, FA, MUFA, PUFA, and CMM prevalence. This finding is consistent with our initial hypothesis that lipid metabolites could act as intermediates linking diet to CMM, providing novel insights to inform future biological mechanisms and longitudinal studies.

Our research found that the intake of VE, FA, PUFA and MUFA is negatively associated with the risk of CMM. This finding is consistent with the results of previous studies. A growing body of evidence shows that VE intake is associated with a lower risk of CMD. For example, studies [20,21,22] reported an association between higher VE intake and a reduced risk of CVD events. In vitro studies confirmed that VE may exert its protective effects through antioxidative and anti-inflammatory mechanisms, as oxidative stress and inflammation are critical factors in the pathogenesis of CVD [23,24]. However, there is ongoing debate regarding the impact of VE supplementation on CVD mortality; a randomized controlled trial (RCT) [25] indicated that VE supplementation does not significantly reduce all-cause mortality in CVD patients. A meta-analysis indicated that VE significantly improves levels of hemoglobin A1c, fasting insulin, and HOMA-IR in patients with Type 2 Diabetes Mellitus (T2DM) [26]. In in vivo studies, it was found that VE supplementation can reduce the risk of diabetes through reduction in oxidative stress and the improvement of endothelial dysfunction [27]. However, there are still contradictory outcomes regarding the protective effect of supplementing VE on diabetes, as an RCT found that VE supplementation does not confer protection against diabetes [28]. Fatty acid intake is closely associated with CVD. Fatty acids exhibit diverse functions depending on their types. They are broadly classified into SFA and UFA. The UFA are further classified into MUFA and PUFA, and they play crucial roles in anti-inflammatory and lipid-lowering activities [29]. Evidence indicates that intake of omega-3 and omega-6 PUFAs can reduce the risk of CVD and cardiovascular mortality [30,31,32,33]. The PREDIMED study also found that replacing SFA with MUFA and PUFA in the diet significantly reduces the risk of CVD [34]. Recent studies show that replacing palmitic acid with oleic acid can improve insulin sensitivity and protect against diabetes [35]. A cross-sectional study found that regular consumption of foods rich in MUFA and PUFA, like those in the Mediterranean diet, benefits individuals with hyperglycemia, metabolic syndrome, and diabetes [36]. Collectively, these findings underscore the intricate relationship between dietary vitamin and fatty acid intake and disease.

Untargeted metabolomics provides a comprehensive characterization of disease-related metabolites. In our case–control study, significant changes in the metabolic profile of patients with CMM were observed. Specifically, alterations were noted in glycerides, glycerophospholipids, sphingolipids, and sterols, suggesting that lipid metabolic disorders play a role in the pathogenesis of CMM. Differential metabolite analysis revealed that among the metabolic profiles of CMM patients, glycerolipids (including DG and TG) exhibited the most significant changes, followed by glycerophospholipids. Numerous studies have confirmed that metabolic profile changes significantly in cardiovascular CVD. For instance, an untargeted plasma metabolomics study of patients with both diabetes and CVD found significant alterations in glycerophospholipids (e.g., PC (O-36:1), PE (O-36:4), PC (28:0)), sterols (e.g., CholesterylEster (CE) (18:0)), and glycerolipids (e.g., DG (16:0–22:5)) [37]. Additionally, a prospective cohort study using lipid metabolomics identified sphingolipid Ceramide (Cerd) (18:1/18:0) as a risk factor for CVD [38]. Similarly, a prospective study conducted in China revealed that TG, DG, and LPC have specific associations with T2DM. Among these, LPC O-16:0 and LPC O-18:0 have been validated to potentially contribute to the development of T2DM via reduced insulin secretion [39]. These findings underscore the association between lipid metabolites and CMD. Furthermore, we found that glycerolipids (DG and TG) are elevated in patients with CMM. This observation is consistent with currently recognized risk factors for cardiovascular metabolic disease, including elevated high-density lipoprotein, decreased low-density lipoprotein, and increased TG. Consistent with these findings, a study investigating the impact of lipid carbon chain length and double bond content on insulin sensitivity reported that TG (52:1) is associated with an increased risk of T2DM [40]. This observation was further validated in a prospective study, which demonstrated that the combination of TG (52:1) with FA (16:0) and FA (18:0), namely, TG (52:1-FA16:0) and TG (52:1-FA18:0), is closely linked to T2DM [41]. Consistent with these findings, our study also observed elevated levels of the metabolites TG (52:1-FA16:0) and TG (52:1-FA18:0) in the disease group. Previous research has suggested that FA (16:0) and FA (18:0) may impair insulin sensitivity, thereby increasing the risk of diabetes [42]. Similarly, in the context of CVD, elevated levels of TG (54:2) have been identified as a strong predictor of cardiovascular disease [43]. In line with these findings, our study observed increased levels of TG (54:2) in combination with FA (16:0), (18:0), (18:1), (18:2), (20:0), and (20:1) in the disease group. Notably, the studies that have reported findings consistent with ours have primarily focused on CVD and diabetes, with limited evidence directly addressing CMM. Further validation is therefore required to elucidate the specific roles of these lipid species in the pathogenesis of CMM.

Through our mediation analysis, we found that lipid metabolites may help explain the observed associations between dietary vitamin and fatty acid intake and CMM risk. Our findings suggest that triglycerides (TG and DG) are the predominant metabolites showing mediation in the relationship between VE intake and CMM prevalence, followed by glycerophospholipids (including LysoPC, LysoPE, LysoPG, PC, PE, PI, PG, and PS), sphingolipids (such as Cer and Hexosylceramide (HexCerd)), and sterol lipids (like CE). Specifically, among the triglycerides, TG (56:9-FA22:6) and TG (56:5-FA22:5) account for 28.5% and 26.2%, respectively. Among the glycerophospholipids, PI (18:1/20:4) has the highest proportion at 29.4%. Existing studies have demonstrated that VE intake is associated with several lipid classes, including PCs, phosphatidylinositols (PIs), triacylglycerols (TAGs), sphingolipids (SEs), and DAGs. Furthermore, changes in these lipid metabolites suggest that the intake of VE may contribute to reduced risk of CVD [44]. A systematic review and meta-analysis found that individuals with T2DM exhibited elevated levels of DAGs, TGs, Cer, PEs, and selected acylcarnitines. These lipid metabolites were associated with an increased risk of T2DM [45]. Among them, ceramides were particularly associated with glucose metabolism disorders [46]. Consistent with our findings, our research also revealed that VE intake is linked to glycerides, glycerophospholipids, and sphingolipids, which in turn are associated with CMM. In the context of PUFA, our analysis showed that glycerolipids account for 57.1% of the relationship between PUFA intake and disease, followed by glycerophospholipids. Notably, TG (60:11-FA22:6) and PE (18:0/22:6) account for 24% and 24.4%, respectively. This finding is supported by an RCT that substituted saturated fatty acid-rich foods with unsaturated fatty acid-rich foods. The study demonstrated that PUFA-rich diets significantly altered plasma lipid metabolites, including sphingolipids (Cer, Hcer) and glycerolipids (TG, DG), which are closely associated with CMD [11]. Regarding MUFA and FA intake, glycerophospholipids dominate as intermediates in disease mediation. Specifically, PI (18:1/20:4) is the primary mediator in MUFA consumption and CMM prevalence, accounting for 29.4% of intermediates. Studies have shown that a diet rich in MUFA significantly reduces levels of DAG (14:0, 15:0, 16:0, 18:0, 22:4), TG (14:1, 16:0, 17:0, 18:0, 20:0), SM (14:0, 18:0), and HCER (18:1) [11]. However, fewer studies have reported on glycerophospholipids. In the regulation of FA intake and CMM incidence, PI (18:1/18:2) is the main mediator, accounting for 32.26% of intermediates. In summary, glycerophospholipids play a significant role in mediating the relationship between FA and MUFA intake and CMM incidence, while glycerolipids are more prominent in the intake of VE and PUFA-mediated links.

Given the high prevalence and mortality rate of CMM, the prevention of CMM is of critical importance. To address this challenge, we developed and validated a predictive model for CMM risk. Our model integrates metabolomics information with dietary vitamins and fatty acid intake. This combination not only enhances the predictive performance but also achieves remarkable classification accuracy. Our findings suggest that this integrated approach may offer insights into early detection and prevention strategies for CMM, potentially contributing to improved public health outcomes.

However, the current study has several limitations. Firstly, dietary intake was assessed using a semi-quantitative food frequency questionnaire (SFFQ), which is susceptible to multiple uncontrollable factors and prone to self-reporting bias and recall bias. Moreover, we did not measure corresponding nutritional biomarkers that could objectively validate the reported intakes. The inclusion of such biomarkers would have strengthened the reliability of our exposure assessment. Consequently, the estimation of dietary vitamin and fatty acid intake could be imprecise. Secondly, our cohort consisted of residents from Ningxia Province, predominantly of Han and Hui ethnicities. Therefore, the generalizability of our findings to other populations may be limited. Additionally, this study employed a cross-sectional design without longitudinal follow-up, which limits our ability to establish causal relationships between dietary vitamin and fatty acid intake, lipid metabolites, and disease outcomes. Taken together, these limitations suggest the need for future prospective studies with more rigorous methodologies to validate and extend our findings.

In summary, the present study suggests that higher intake of VE, FA, PUFA, and MUFA is associated with a lower prevalence of CMM. Plasma metabolism is frequently altered in patients with CMM, with the most significant changes observed in triglycerides, followed by glycerophospholipids. Lipid metabolites may serve as potential factors involved in the associations between VE, FA, PUFA, and MUFA intake and CMM risk. Additionally, dietary vitamin and fatty acid intake are linked to CMM risk, possibly accompanied by alterations in different lipid classes. Overall, metabolomics offers valuable insights into potential biological pathways underlying the associations between dietary vitamin and fatty acid intake and CMM.

## 4. Methods

### 4.1. Study Population

Our study was conducted using a case–control design that included 200 patients with CMM and 200 healthy controls matched by sex and age from the Ningxia general population cohort (project number: 2017YFC0907204). The cohort was established between March 2018 and August 2019, drawing from the natural population of individuals aged 35 to 74 years residing in four townships within Pingluo County and Qingtongxia City, Ningxia. By August 2019, baseline surveys and blood sample collections had been completed for 15,802 participants from the rural natural population (Supplementary Figure S1 displays the flow chart). The study was approved by the Ethics Committee of Ningxia Medical University (approval number 2018-012) and all participants provided written informed consent.

### 4.2. Diagnostic Criteria for CMM

CMM specifically denotes the concurrent presence of two or more CMDs, such as diabetes, IHD, stroke, and other CVDs [2,3]. T2DM was diagnosed based on a fasting plasma glucose level ≥ 7.0 mmol/L [47]. Hypertension was defined as SBP ≥ 140 mmHg or DBP ≥ 90 mmHg [48]. Diagnoses of IHD, stroke, and other CVDs were confirmed based on physician diagnosis, ECG evidence, or reliable self-reported medical history [49].

### 4.3. Inclusion and Exclusion Criteria

The case group consisted of individuals with a current clinical diagnosis of CMM, identified from a general population cohort aged 35 to 75 years. The following individuals were excluded: (1) Pregnant or breastfeeding women. (2) Individuals with severe gastrointestinal diseases. (3) Individuals with severe psychiatric disorders or severe infectious diseases. (4) Individuals with autoimmune diseases or other malignancies.

The control group was selected from the general population cohort and matched to cases at a 1:1 ratio by sex and age. The following individuals were excluded: (1) Pregnant or breastfeeding women. (2) Individuals with recent severe infectious diseases. (3) Individuals with psychiatric disorders. (4) Individuals suffering from other conditions that could significantly affect their current health status.

### 4.4. Data Collection

#### 4.4.1. Demographic Information

Baseline data of the participants, including sex, age, ethnicity, marital status, educational level, smoking status, alcohol consumption, physical activity, and medical history, were collected using a standardized questionnaire. Data were collected through face-to-face interviews conducted by trained investigators to ensure consistency and accuracy.

#### 4.4.2. Assessment of Dietary Vitamins and Fatty Acid

In our study, we assessed participants’ dietary intake using a SFFQ that had been validated for reliability and validity [50]. The SFFQ included 15 food categories, comprising a total of 69 specific food items. Each food item provided five frequency options: once per day, 4–6 times per week, 1–3 times per month, and never consumed. We then calculated the average intake of energy, vitamins and fatty acids based on the SSFQ data. Specifically, daily energy and dietary vitamin and fatty acid intake for each individual were determined by multiplying the average single-serving food consumption by the frequency weight and nutrient content per gram of food, using the Chinese Food Composition Table (2023 edition) and the Flying Nutrition Calculator (v.2.7.5 standard version-k).

#### 4.4.3. Anthropometric Examination

Body weight and height were measured using a calibrated electronic scale. Subsequently, BMI was calculated based on these measurements. Participants were instructed to rest quietly for 15 min. After this rest period, SBP and DBP were measured on the right upper arm using an Omron (Kyoto, Japan) calibrated electronic blood pressure monitor. Two measurements were taken for each participant, and the average value was used as the final blood pressure reading.

#### 4.4.4. Blood Collection

Participants were instructed to fast for 8–12 h prior to blood collection and biochemical measurements. Venous blood samples were collected using a disposable blood collection device. Blood samples were collected into two types of vacuum tubes: one containing an anticoagulant for plasma separation, and the other being plain tubes without additives for serum separation. Approximately one hour after collection, the blood samples were processed. Both types of samples were centrifuged at 3000 rpm for 10 min at 4 °C. The resulting supernatants (plasma and serum) were carefully collected and stored at −80 °C for subsequent analysis.

#### 4.4.5. Biochemical Measurements

Biochemical measurements were performed using a fully automated biochemical analyzer (Mindray BS-430, Shenzhen, China). FBG, uric acid (UA), HDL-C, low-density lipoprotein-cholesterol (LDL-C), TG and total cholesterol (TC) were measured using this instrument.

### 4.5. Plasma Lipid Metabolomics Analysis

#### 4.5.1. Sample Preparation

Firstly, 20 microliters of plasma was transferred to a 1.5 mL EP tube (Corning Incorporated, Corning, NY, USA). Then, 20 μL of isotope internal standard mixture and 180 μL of isopropanol containing 10 mM ammonium acetate were added to the tube. The mixture was vortexed at 1500 rpm for 5 min. Subsequently, the samples were centrifuged at 13,000× *g* for 15 min. Finally, 150 μL of the supernatant was carefully transferred to a 96-well plate for subsequent analysis.

#### 4.5.2. Ultra-High-Performance Liquid Chromatography Coupled with Triple-Quadrupole Mass Spectrometry

Analyses were performed using a Thermo Scientific Ultimate 3000 liquid chromatography system (Thermo Scientific, Dreieich, Germany) coupled with a TSQ Endura MD triple quadrupole mass spectrometer (Thermo Scientific, Waltham, MA, USA). Chromatographic separation was achieved on an ACQUITY UPLC^®^ HSS C18 column (particle size 1.8 μm, dimensions 2.1 mm × 100 mm). The mobile phase comprised two components: solvent A (60% acetonitrile and 40% water containing 5 mM ammonium acetate) and solvent B (90% isopropanol and 10% acetonitrile containing 5 mM ammonium acetate), delivered at a flow rate of 0.3 mL/min. The LC gradient program was as follows: 0.0 min, 30% B; 3.0 min, 70% B; 12.5 min, 95% B; 12.6 min, 30% B. Mass spectrometry was performed using a heated electrospray ionization (HESI) source. The instrument parameters were optimized as follows: ionization voltage of +3.5 kV for positive ions and −3.0 kV for negative ions, ion source temperature of 350 °C, sheath gas flow rate of 40 arbitrary units (Arb), auxiliary gas flow rate of 15 Arb, and collision gas pressure of 2.0 mTorr. Data acquisition was carried out in selected reaction monitoring (SRM) mode. To ensure analytical reproducibility and monitor system stability, a pooled quality control (QC) sample was prepared by combining equal volumes of randomly selected plasma samples. Prior to the commencement of formal sample analysis, multiple consecutive injections of the QC sample were performed to allow the instrument to reach a stable state and achieve equilibration. Subsequently, one QC sample was injected after approximately every 30 study samples, resulting in a total of 24 QC injections spanning the entire analytical batch. Additionally, the mass spectrometer automatically performed internal calibration after every seven sample analyses to maintain measurement accuracy. System performance was assessed by monitoring the coefficient of variation (CV%) of lipid feature peak areas in the QC samples. Only lipid features exhibiting CV% < 20% in the QC samples were considered sufficiently reproducible and included in subsequent data analysis.

#### 4.5.3. Data Analysis

Mass spectrometry data were processed using TraceFinder 5.1 software (Thermo Scientific, Waltham, MA, USA), and lipid quantification was performed using the stable isotope lipid internal standard single-point correction method. Metabolites comments were made using public databases, including HMDB (https://hmdb.ca/, accessed on 15 June 2024), KEGG (https://www.kegg.jp/kegg/compound/, accessed on 15 June 2024), LIPIDMAPS (https://lipidmaps.org/, accessed on 16 June 2024), PubChem (https://pubchem.ncbi.nlm.nih.gov/, accessed on 16 June 2024), and ChEBI (https://www.ebi.ac.uk/chebi/, accessed on 17 June 2024).

### 4.6. Statistical Analysis

Firstly, the metabolic data were analyzed using SIMCA-P+ version 14.0 (Umetrics, Umea, Sweden). Differential metabolites between the two groups were identified using OPLS-DA and model validation, with metabolites selected based on a VIP score > 1 and a *p*-value ≤ 0.05. Next, the distribution characteristics of the data were evaluated. Parametric statistical tests were applied to data that followed a normal distribution, while non-parametric tests were used for data with a non-normal distribution. For non-normally distributed variables, appropriate transformations were applied when necessary to approximate normality. Associations between dietary vitamin and fatty acid intake and differential metabolites were assessed using Pearson’s correlation coefficient. For logistic regression analysis of dietary vitamin and fatty acid intake and disease risk, nutrient intakes were energy-adjusted using the residual method: each intake was regressed on total energy intake, and the residuals were used as the adjusted exposure. The analysis was conducted under three models, each adjusting for different covariates: Model 1 was the unadjusted crude model; Model 2 was adjusted for sex, age, and physical activity; Model 3 was adjusted for sex, age, physical activity, education, nation, marital status, smoking status and alcohol consumption. All statistical analyses were performed using IBM SPSS 21 software (SPSS Inc., Chicago, IL, USA). Following that, mediation analysis was performed using the “mediation” R package (version 4.5.0) to assess the extent to which metabolites contributed to the effect of dietary vitamin and fatty acid intake on the risk of CMM. Finally, the AUROC was calculated using the “pROC” R package (version 1.18.5) to evaluate the predictive performance of combinations of vitamin or fatty acid intake and metabolites in CMM risk models.

## Figures and Tables

**Table 1:** Baseline characteristics of the study.

Characteristics	CMM ^1^	Healthy	*p*
(median (P25, P75))			
N	200	200	
Age, y	66 (61, 69)	65 (61, 69)	0.795
Sex, n (%)			
Female	117 (58.5%)	117 (58.5%)	1.000
Male	83 (41.5%)	83 (41.5%)	
Nation, n (%)			
the Han nationality	97 (48.5%)	93 (46.5%)	0.689
the Hui nationality	103 (51.5%)	107 (53.5%)	
Smoking, n (%)	27 (13.5%)	33 (16.5%)	0.401
Alcohol intake, n (%)	16 (8%)	30 (15%)	0.028
Marriage, n (%)			
Married	174 (87%)	173 (86.5%)	0.883
Others	26 (13%)	27 (13.5%)	
Physical activity, n (%)			
High	84 (42%)	89 (44.5%)	0.703
Low	67 (33.5%)	65 (32.5%)	
SBP (mmHg)	145 (135.00, 158.25)	133 (122.00, 146.50)	<0.001
DBP (mmHg)	88 (79.00, 96.00)	80 (72, 88)	<0.001
BMI (kg/m^2^)	25.85 (24.10, 28.30)	23.85 (22.00, 26.15)	<0.001
FBG (mmol/L)	7.13 (5.50, 9.70)	5.30 (4.90, 5.77)	<0.001
TC (mmol/L)	4.91 (4.13, 5.62)	4.80 (4.01, 5.34)	0.254
TG (mmol/L)	1.72 (1.22, 2.67)	1.31 (0.90, 1.98)	<0.001
HDL-c (mmol/L)	1.17 (0.95, 1.45)	1.30 (1.12, 1.55)	<0.001
LDL-c (mmol/L)	2.93 (2.25, 3.62)	2.91 (2.31, 3.44)	0.872
UA (μmol/L)	281.5 (231.25, 349.00)	270 (218.50, 325.00)	0.053
Comorbidity			
Hypertension, n%	182 (91%)	0 (0%)	<0.001
Diabetes, n%	130 (65%)	0 (0%)	<0.001
AMI, n%	34 (17%)	0 (0%)	<0.001
Angina, n%	24 (12%)	0 (0%)	<0.001
Stroke, n%	39 (19.5%)	0 (0%)	<0.001
Others, n%	29 (14.5%)	0 (0%)	<0.001
Diabetes + Hypertension, n%	118 (59%)	0 (%)	<0.001

Abbreviation: CMM ^1^, cardiometabolic multimorbidity (defined as the co-occurrence of two or more cardiometabolic conditions such as diabetes, ischemic heart disease, stroke, and other cardiovascular diseases); SBP, systolic pressure; DBP, diastolic blood pressure; BMI, body mass index; FBG, fasting blood glucose; TC, total cholesterol; TG, triglycerides; HDL-c, high-density lipoprotein cholesterol; LDL-c, low-density lipoprotein cholesterol; UA, uric acid; AMI, acute myocardial infarction.

**Table 2:** The dietary vitamins and fatty acid intake in the study population.

Vitamins and Fatty Acids	CMM	Healthy	*p*
	N = 200	N = 200	
	Median (P25, P75)	Median (P25, P75)	
Energy (kcal/d)	1876.86 (1454.26, 2268.92)	1977.12 (1568.98, 2564.04)	0.016
VA (μgRAE/d)	1992.01 (100.64, 9044.21)	4568.60 (180.76, 9258.70)	0.060
VB1 (mg/d)	0.72 (0.46, 0.97)	0.79 (0.51, 1.10)	0.028
VB2 (mg/d)	1.10 (0.39, 2.57)	1.54 (0.45, 2.89)	0.076
Niacin (mg/d)	13.05 (7.70, 23.33)	16.66 (7.76, 27.26)	0.041
VB6 (mg/d)	0.14 (0.09, 0.27)	0.18 (0.09, 0.31)	0.134
Folic acid (μg/d)	113.03 (65.40, 189.56)	126.61 (76.07, 208.16)	0.041
VC (mg/d)	39.13 (27.08, 73.09)	54.76 (27.08, 86.67)	0.033
VD (μg/d)	1.07 (0.87, 3.86)	1.70(0.96, 6.87)	0.072
VE (mg/d)	129.40 (81.49, 190.41)	145.79 (100.18, 224.79)	0.007
FA (g/d)	59.79 (41.87, 89.56)	67.25 (44.62, 96.25)	0.039
SFA (g/d)	10.36 (7.33, 14.77)	11.19 (8.05, 17.89)	0.036
MUFA (g/d)	23.14 (16.75, 33.79)	25.98 (17.79, 35.86)	0.033
PUFA (g/d)	24.06 (16.19, 35.63)	26.46 (17.06, 37.76)	0.069

Abbreviation: VA, vitamin A; VD, vitamin D; VB1, vitamin B1; VB2, vitamin B2; VB6, vitamin B6; VC, vitamin C; VE, vitamin E; FA, total fatty acids; SFA, saturated fatty acids; MUFA, monounsaturated fatty acids; PUFA, polyunsaturated fatty acids.

**Table 3:** Associations of dietary vitamin and fatty acid intake with CMM.

Factors	Model 1 ^1^			Model 2 ^2^			Mode 3 ^3^		
	OR (95%CI) ^4^	*p* value	FDR	OR (95%CI) ^5^	*p* value	FDR	OR (95%CI) ^6^	*p* value	FDR
VA	0.88 (0.73–1.06)	0.175	0.207	0.89 (0.73–1.07)	0.219	0.258	0.89 (0.72–1.09)	0.253	0.299
VB1	0.81 (0.68–0.98)	0.028	0.061	0.82 (0.68–0.99)	0.042	0.090	0.82 (0.67–0.99)	0.045	0.098
VB2	0.87 (0.72–1.04)	0.127	0.166	0.87 (0.72–1.06)	0.164	0.214	0.87 (0.71–1.07)	0.182	0.237
Niacin	0.83 (0.69–1.00)	0.047	0.088	0.84 (0.69–1.01)	0.067	0.124	0.83 (0.68–1.02)	0.074	0.137
VB6	0.92 (0.76–1.11)	0.373	0.405	0.93 (0.77–1.12)	0.413	0.447	0.92 (0.76–1.12)	0.411	0.411
Folic acid	0.86 (0.72–1.04)	0.115	0.165	0.87 (0.72–1.05)	0.141	0.204	0.87 (0.71–1.06)	0.154	0.223
VC	0.86 (0.71–1.03)	0.104	0.165	0.86 (0.71–1.04)	0.131	0.204	0.86 (0.70–1.05)	0.136	0.222
VD	0.93 (0.7–1.12)	0.436	0.436	0.93 (0.78–1.13)	0.470	0.470	0.90 (0.74–1.09)	0.276	0.299
VE	0.75 (0.61–0.90)	0.002	0.032	0.74 (0.61–0.89)	0.002	0.027	0.74 (0.61–0.90)	0.002	0.031
FA	0.78 (0.65–0.94)	0.008	0.038	0.78 (0.64–0.94)	0.009	0.041	0.77 (0.64–0.93)	0.008	0.037
PUFA	0.80 (0.66–0.96)	0.016	0.045	0.79 (0.66–0.95)	0.014	0.045	0.79 (0.66–0.96)	0.015	0.049
MUFA	0.78 (0.65–0.94)	0.009	0.038	0.78 (0.65–0.94)	0.009	0.041	0.77 (0.64–0.94)	0.009	0.037
SFA	0.80 (0.66–0.96)	0.017	0.045	0.80 (0.66–0.97)	0.023	0.059	0.79 (0.65–0.96)	0.019	0.051

^1^ Crude model. ^2^ Adjusted for age, sex, physical activity. ^3^ Adjusted for age, sex, physical activity, education, nation, marital status, smoking status, and alcohol consumption. ^4–6^ The relationships between dietary vitamins and fatty acid intake and CMM under adjusting different covariate models.

## Data Availability

The data that support the findings of this study are not openly available due to reasons of sensitivity and are available from the corresponding author upon reasonable request.

## Supplementary Materials

The following supporting information can be downloaded at: https://www.mdpi.com/article/10.3390/ijms262311477/s1.
