# Supplementary material for: Vitamin E and Fatty Acid Intake and Cardiometabolic Multimorbidity Risk: The Mediating Role of Plasma Lipid Metabolites
Source: Int J Mol Sci. 2025 Nov 27;26(23):11477. doi: 10.3390/ijms262311477 (PMC12691755; doi:10.3390/ijms262311477)
Supplement: Supplementary file 1 [file ijms-26-11477-s001.zip › Supplementary figure.pdf]

Supplementary Figure S1

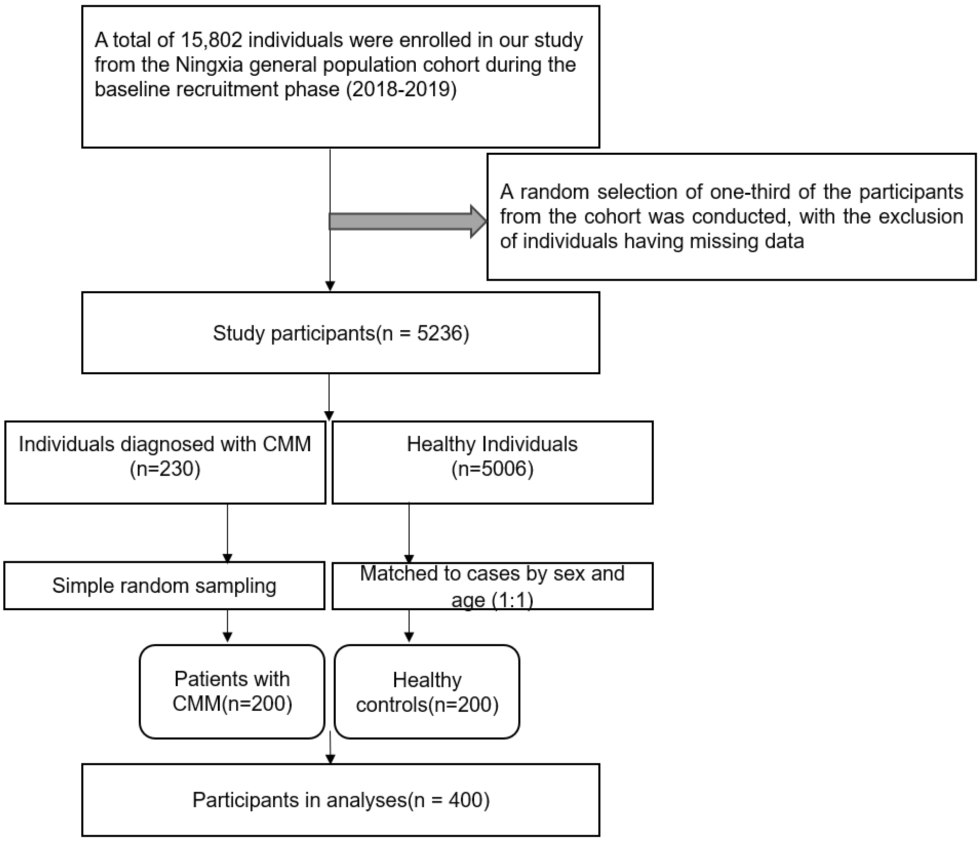

Supplementary Figure S1: Flow chart of participants in the Ningxia general population cohort study.
