# Supplementary material for: Vitamin E and Fatty Acid Intake and Cardiometabolic Multimorbidity Risk: The Mediating Role of Plasma Lipid Metabolites
Source: Int J Mol Sci. 2025 Nov 27;26(23):11477. doi: 10.3390/ijms262311477 (PMC12691755; doi:10.3390/ijms262311477)
Supplement: Supplementary file 1 [file ijms-26-11477-s001.zip › Supplementary table.pdf]

Supplementary Table S1 Different Lipid metabolites in CMM and control populations

Number	Metabolite	VIP	P	FC	HMDB	CMM vs Control
1	CE (20:5)	1.07	0.0004	1.31	LMST01020015	up
2	Cer (d18:0/22:1)	1.14	0.0000	1.25		up
3	Cer (d18:1/18:0)	1.05	0.0000	1.40	LMSP02010006	up
4	DG (16:0/18:1)	1.32	0.0000	1.35	LMGL02010004	up
5	DG (16:0/18:2)	1.03	0.0000	1.21	LMGL02010027	up
6	DG (16:0/18:3)	1.04	0.0000	144	LMGL02010474	up
7	DG (18:0/18:1)	1.37	0.0000	1.30	LMGL02010043	up
8	DG (18:0/18:2)	1.29	0.0000	1.26	LMGL02010050	up
9	HexCer (d18:1/24:1)	1.02	0.0019	0.91		down
10	LacCer (d18:1/14:0)	1.09	0.0000	0.85	LMSP0501AB12	down
11	LacCer (d18:1/16:0)	1.01	0.0000	0.89	LMSP0501AB03	down
12	LacCer (d18:1/24:1)	1.22	0.0000	0.86		down
13	LysoPC (14:0)	1.08	0.0000	1.22		up
14	LysoPC (19:0)	1.22	0.0001	0.88		down
15	LysoPC (20:0)	1.21	0.0000	0.88		down
16	LysoPC (20:1)	1.40	0.0001	0.88	LMGP01050047	down
17	LysoPC (20:5)	1.27	0.0000	1.34	LMGP01050050	up
18	LysoPC (22:0)	1.30	0.0000	0.85		down
19	LysoPC (22:1)	1.14	0.0003	0.87		down
20	LysoPC (22:5)	1.07	0.0021	1.17		up
21	LysoPC (24:0)	1.09	0.0000	0.89		down
22	LysoPC (24:1)	1.58	0.0000	0.83		down
23	LysoPC (O-22:0)	1.01	0.0046	0.94		down
24	LysoPC (P-18:0)	1.16	0.0004	0.89		down
25	LysoPE (20:2)	1.18	0.0001	0.85		down
26	LysoPE (20:5)	1.13	0.0020	1.24		up
27	LysoPE (22:0)	1.19	0.0002	0.92		down
28	LysoPG (18:0)	1.22	0.0000	1.28		up
29	PC (16:0/20:5)	1.25	0.0000	1.26	LMGP01010633	up
30	PC (16:1/18:2)	1.21	0.0011	0.91	LMGP01010690	down
31	PC (18:0/20:5)	1.04	0.0023	1.19	LMGP01010805	up
32	PC (18:1/18:1)	1.54	0.0019	0.91	LMGP01010836	down
33	PC (18:1/18:2)	1.74	0.0000	0.87	LMGP01010893	down
34	PC (18:1/20:1)	1.31	0.0043	0.90	LMGP01010843	down
35	PC (18:1/20:2)	1.28	0.0143	0.92	LMGP01011608	down
36	PC (18:1/20:3)	1.27	0.0013	0.91	LMGP01010904	down
37	PC (18:1/20:4)	1.24	0.0001	0.91	LMGP01010905	down
38	PC (18:1/22:4)	1.29	0.0003	0.88	LMGP01011613	down
39	PC (18:1/22:5)	1.10	0.0000	0.86	LMGP01010846	down
40	PC (18:1/22:6)	1.15	0.0000	0.87	LMGP01010847	down
41	PC (18:2/16:1)	1.21	0.0011	0.91		down
42	PC (18:2/18:2)	1.29	0.0000	0.87	LMGP01010921	down

43	PC (18:2/20:1)	1.50	0.0000	0.82	LMGP01010942	down
44	PC (18:2/20:2)	1.39	0.0000	0.83	LMGP01011632	down
45	PC (18:2/20:3)	1.08	0.0349	0.93	LMGP01011633	down
46	PC (18:2/22:4)	1.24	0.0013	0.89	LMGP01011639	down
47	PC (18:2/22:6)	1.08	0.0001	0.88	LMGP01010947	down
48	PC (20:0/18:1)	1.24	0.0211	0.93		down
49	PC (20:0/20:3)	1.07	0.0013	0.90	LMGP01011022	down
50	PC (O-18:0/18:2)	1.12	0.0279	0.93	LMGP01020203	down
51	PC (O-18:1/18:2)	1.55	0.0000	0.87	LMGP01020262	down
52	PC (P-16:0/18:2)	1.26	0.0003	0.91	LMGP01030008	down
53	PC (P-16:0/20:2)	1.09	0.0007	0.90	LMGP01030038	down
54	PC (P-18:0/18:2)	1.22	0.0008	0.87	LMGP01030058	down
55	PC (P-18:1/18:1)	1.65	0.0000	0.83	LMGP01030137	down
56	PE (16:0/16:0)	1.01	0.0000	1.28		up
57	PE (16:0/20:5)	1.03	0.0048	1.23	LMGP02010968	up
58	PE (18:0/18:1)	1.03	0.0006	1.19	LMGP02010036	up
59	PE (18:0/20:5)	1.17	0.0004	1.35	LMGP02010973	up
60	PE (18:0/22:5)	1.16	0.0009	1.22		up
61	PE (18:0/22:6)	1.30	0.0000	1.27	LMGP02010094	up
62	PE (P-18:1/18:2)	1.06	0.0105	0.90		down
63	PE (P-18:1/22:4)	1.09	0.0059	0.91		down
64	PG (18:0/18:1)	1.21	0.0005	1.25	LMGP04010037	up
65	PG (18:0/18:2)	1.18	0.0007	1.17	LMGP04010876	up
66	PI (14:0/18:0)	1.10	0.0000	1.28	LMGP06010859	up
67	PI (18:0/22:5)	1.11	0.0017	1.16		up
68	PI (18:1/18:1)	1.35	0.0003	0.82	LMGP06010966	down
69	PI (18:1/18:2)	1.71	0.0000	0.78	LMGP06010318	down
70	PI (18:1/20:3)	1.59	0.0000	0.78	LMGP06010303	down
71	PI (18:1/20:4)	1.66	0.0000	0.80	LMGP06010599	down
72	PI (18:2/18:2)	1.21	0.0000	0.77	LMGP06010927	down
73	PI (19:0/20:4)	1.08	0.0000	0.83	LMGP06010440	down
74	PS (20:0/18:1)	1.23	0.0001	0.84		down
75	TG (42:0-FA14:0)	1.09	0.0065	1.31		up
76	TG (42:0-FA16:0)	1.09	0.0037	1.47		up
77	TG (42:1-FA14:0)	1.05	0.0106	1.24		up
78	TG (42:1-FA16:0)	1.05	0.0098	1.45		up
79	TG (42:1-FA16:1)	1.08	0.0096	1.35		up
80	TG (42:1-FA18:1)	1.00	0.0050	1.39		up
81	TG (44:0-FA14:0)	1.16	0.0015	1.52		up
82	TG (44:0-FA16:0)	1.16	0.0033	1.48		up
83	TG (44:1-FA14:0)	1.13	0.0141	1.30		up
84	TG (44:1-FA16:0)	1.15	0.0046	1.42		up
85	TG (44:1-FA16:1)	1.17	0.0152	1.28		up
86	TG (44:1-FA18:1)	1.12	0.0042	1.34		up

87	TG (44:2-FA16:0)	1.11	0.0014	1.40	up
88	TG (44:2-FA18:2)	1.15	0.0046	1.34	up
89	TG (46:0-FA14:0)	1.21	0.0006	1.63	up
90	TG (46:0-FA16:0)	1.22	0.0008	1.62	up
91	TG (46:0-FA18:0)	1.11	0.0045	1.34	up
92	TG (46:1-FA14:0)	1.23	0.0021	1.43	up
93	TG (46:1-FA16:0)	1.20	0.0041	1.43	up
94	TG (46:1-FA16:1)	1.20	0.0035	1.42	up
95	TG (46:1-FA18:0)	1.14	0.0036	1.39	up
96	TG (46:1-FA18:1)	1.22	0.0026	1.32	up
97	TG (46:2-FA14:0)	1.25	0.0039	1.29	up
98	TG (46:2-FA16:0)	1.21	0.0086	1.28	up
99	TG (46:2-FA18:2)	1.23	0.0018	1.28	up
100	TG (46:3-FA14:0)	1.22	0.0018	1.43	up
101	TG (46:3-FA16:0)	1.27	0.0021	1.35	up
102	TG (46:3-FA18:2)	1.12	0.0390	1.16	up
103	TG (46:3-FA18:3)	1.24	0.0009	1.47	up
104	TG (46:4-FA18:2)	1.00	0.0113	1.20	up
105	TG (48:0-FA14:0)	1.26	0.0003	1.65	up
106	TG (48:0-FA16:0)	1.34	0.0001	1.62	up
107	TG (48:0-FA18:0)	1.25	0.0003	1.64	up
108	TG (48:1-FA14:0)	1.33	0.0003	1.41	up
109	TG (48:1-FA16:0)	1.30	0.0006	1.44	up
110	TG (48:1-FA16:1)	1.26	0.0008	1.50	up
111	TG (48:1-FA18:0)	1.21	0.0030	1.38	up
112	TG (48:1-FA18:1)	1.32	0.0004	1.38	up
113	TG (48:2-FA14:0)	1.36	0.0006	1.33	up
114	TG (48:2-FA16:0)	1.31	0.0013	1.34	up
115	TG (48:2-FA16:1)	1.16	0.0169	1.28	up
116	TG (48:2-FA18:0)	1.08	0.0162	1.22	up
117	TG (48:2-FA18:2)	1.39	0.0001	1.36	up
118	TG (48:3-FA14:0)	1.36	0.0005	1.37	up
119	TG (48:3-FA16:0)	1.31	0.0006	1.44	up
120	TG (48:3-FA18:3)	1.34	0.0002	1.51	up
121	TG (48:4-FA14:0)	1.21	0.0105	1.28	up
122	TG (48:4-FA16:1)	1.20	0.0221	1.23	up
123	TG (48:4-FA18:1)	1.05	0.0445	1.15	up
124	TG (48:4-FA18:3)	1.21	0.0128	1.27	up
125	TG (48:4-FA20:4)	1.21	0.0040	1.33	up
126	TG (49:0-FA16:0)	1.03	0.0304	1.32	up
127	TG (49:0-FA17:0)	1.02	0.0320	1.30	up
128	TG (49:1-FA16:0)	1.10	0.0340	1.25	up
129	TG (49:2-FA16:0)	1.14	0.0399	1.18	up
130	TG (49:2-FA18:2)	1.08	0.0224	1.17	up

131	TG (49:3-FA16:0)	1.14	0.0160	1.26	up
132	TG (49:3-FA18:3)	1.06	0.0200	1.27	up
133	TG (50:0-FA14:0)	1.23	0.0003	1.53	up
134	TG (50:0-FA16:0)	1.34	0.0001	1.73	up
135	TG (50:0-FA18:0)	1.33	0.0000	1.74	up
136	TG (50:1-FA14:0)	1.37	0.0000	1.45	up
137	TG (50:1-FA16:0)	1.45	0.0000	1.41	up
138	TG (50:1-FA16:1)	1.33	0.0002	1.56	up
139	TG (50:1-FA18:0)	1.35	0.0001	1.52	up
140	TG (50:1-FA18:1)	1.44	0.0000	1.40	up
141	TG (50:1-FA20:1)	1.23	0.0010	1.27	up
142	TG (50:2-FA14:0)	1.17	0.0052	1.19	up
143	TG (50:2-FA16:0)	1.46	0.0001	1.33	up
144	TG (50:2-FA16:1)	1.25	0.0040	1.26	up
145	TG (50:2-FA18:0)	1.43	0.0000	1.40	up
146	TG (50:2-FA18:1)	1.24	0.0051	1.21	up
147	TG (50:2-FA18:2)	1.51	0.0000	1.34	up
148	TG (50:2-FA20:2)	1.26	0.0005	1.32	up
149	TG (50:3-FA14:0)	1.12	0.0079	1.14	up
150	TG (50:3-FA16:0)	1.48	0.0000	1.37	up
151	TG (50:3-FA16:1)	1.21	0.0154	1.17	up
152	TG (50:3-FA18:0)	1.39	0.0000	1.46	up
153	TG (50:3-FA18:1)	1.11	0.0297	1.13	up
154	TG (50:3-FA18:2)	1.28	0.0040	1.18	up
155	TG (50:3-FA18:3)	1.50	0.0000	1.58	up
156	TG (50:3-FA20:3)	1.27	0.0002	1.43	up
157	TG (50:4-FA14:0)	1.24	0.0017	1.20	up
158	TG (50:4-FA16:0)	1.34	0.0024	1.32	up
159	TG (50:4-FA16:1)	1.27	0.0089	1.22	up
160	TG (50:4-FA18:1)	1.26	0.0030	1.24	up
161	TG (50:4-FA18:3)	1.35	0.0015	1.30	up
162	TG (50:4-FA20:3)	1.16	0.0120	1.21	up
163	TG (50:4-FA20:4)	1.24	0.0010	1.45	up
164	TG (50:5-FA14:0)	1.23	0.0008	1.30	up
165	TG (50:5-FA16:0)	1.20	0.0075	1.25	up
166	TG (50:5-FA18:2)	1.20	0.0031	1.24	up
167	TG (50:5-FA18:3)	1.23	0.0088	1.23	up
168	TG (50:5-FA20:5)	1.27	0.0001	1.80	up
169	TG (51:2-FA16:0)	1.14	0.0328	1.20	up
170	TG (51:2-FA17:0)	1.08	0.0280	1.20	up
171	TG (51:2-FA18:2)	1.11	0.0068	1.23	up
172	TG (51:3-FA17:0)	1.07	0.0280	1.21	up
173	TG (51:3-FA18:3)	1.03	0.0121	1.30	up
174	TG (52:0-FA16:0)	1.25	0.0001	1.71	up

175	TG (52:0-FA18:0)	1.16	0.0003	1.71	up
176	TG(52:0-FA20:0)	1.32	0.0001	1.55	up
177	TG(52:1-FA16:0)	1.46	0.0000	1.54	up
178	TG (52:1-FA16:1)	1.31	0.0002	1.51	up
179	TG (52:1-FA18:0)	1.43	0.0000	1.52	up
180	TG (52:1-FA18:1)	1.41	0.0000	1.50	up
181	TG (52:1-FA20:0)	1.29	0.0012	1.37	up
182	TG (52:1-FA20:1)	1.30	0.0000	1.41	up
183	TG (52:2-FA14:0)	1.06	0.0044	1.17	up
184	TG (52:2-FA16:0)	1.28	0.0001	1.20	up
185	TG (52:2-FA16:1)	1.33	0.0002	1.30	up
186	TG (52:2-FA18:0)	1.56	0.0000	1.43	up
187	TG (52:2-FA18:1)	1.22	0.0005	1.20	up
188	TG (52:2-FA18:2)	1.60	0.0000	1.45	up
189	TG (52:2-FA20:0)	1.30	0.0008	1.21	up
190	TG (52:2-FA20:1)	1.16	0.0065	1.21	up
191	TG (52:2-FA20:2)	1.41	0.0000	1.40	up
192	TG (52:3-FA14:0)	1.11	0.0078	1.14	up
193	TG (52:3-FA16:0)	1.20	0.0005	1.16	up
194	TG (52:3-FA18:0)	1.51	0.0000	1.55	up
195	TG (52:3-FA18:1)	1.09	0.0042	1.13	up
196	TG (52:3-FA18:2)	1.11	0.0011	1.14	up
197	TG (52:3-FA18:3)	1.47	0.0000	1.72	up
198	TG (52:3-FA20:0)	1.15	0.0010	1.14	up
199	TG (52:3-FA20:1)	1.11	0.0069	1.13	up
200	TG (52:3-FA20:2)	1.27	0.0019	1.18	up
201	TG (52:3-FA20:3)	1.43	0.0000	1.54	up
202	TG (52:4-FA14:0)	1.12	0.0126	1.15	up
203	TG (52:4-FA16:0)	1.41	0.0001	1.21	up
204	TG (52:4-FA18:0)	1.44	0.0001	1.31	up
205	TG (52:4-FA18:1)	1.37	0.0004	1.20	up
206	TG (52:4-FA18:3)	1.47	0.0000	1.29	up
207	TG (52:4-FA20:0)	1.33	0.0001	1.19	up
208	TG (52:4-FA20:3)	1.27	0.0025	1.22	up
209	TG (52:4-FA20:4)	1.35	0.0000	1.52	up
210	TG (52:4-FA22:4)	1.43	0.0003	1.27	up
211	TG (52:5-FA14:0)	1.35	0.0007	1.26	up
212	TG (52:5-FA16:0)	1.47	0.0000	1.31	up
213	TG (52:5-FA18:2)	1.30	0.0005	1.20	up
214	TG (52:5-FA18:3)	1.42	0.0001	1.26	up
215	TG (52:5-FA20:3)	1.15	0.0160	1.14	up
216	TG (52:5-FA20:4)	1.20	0.0047	1.30	up
217	TG (52:5-FA20:5)	1.30	0.0004	1.42	up
218	TG (52:5-FA22:5)	1.52	0.0000	1.35	up

219	TG (52:6-FA14:0)	1.01	0.0439	1.16	up
220	TG (52:6-FA16:0)	1.27	0.0001	1.44	up
221	TG (52:6-FA18:3)	1.24	0.0005	1.37	up
222	TG (52:6-FA20:4)	1.19	0.0113	1.21	up
223	TG (52:6-FA20:5)	1.31	0.0004	1.50	up
224	TG (52:7-FA16:0)	1.15	0.0040	1.52	up
225	TG (52:7-FA20:5)	1.30	0.0004	1.35	up
226	TG (52:7-FA22:6)	1.27	0.0017	1.32	up
227	TG (53:0-FA16:0)	1.02	0.0256	1.22	up
228	TG (53:1-FA16:0)	1.00	0.0137	1.31	up
229	TG (53:2-FA16:0)	1.08	0.0185	1.19	up
230	TG (53:2-FA18:2)	1.07	0.0024	1.31	up
231	TG (53:3-FA16:0)	1.22	0.0024	1.19	up
232	TG (53:4-FA16:0)	1.30	0.0019	1.21	up
233	TG (54:0-FA16:0)	1.27	0.0005	1.34	up
234	TG (54:1-FA16:0)	1.31	0.0002	1.45	up
235	TG (54:1-FA18:0)	1.12	0.0004	1.54	up
236	TG (54:1-FA18:1)	1.19	0.0003	1.55	up
237	TG (54:1-FA20:0)	1.28	0.0005	1.46	up
238	TG (54:2-FA16:0)	1.18	0.0002	1.27	up
239	TG (54:2-FA18:0)	1.18	0.0001	1.27	up
240	TG (54:2-FA18:1)	1.13	0.0004	1.26	up
241	TG (54:2-FA18:2)	1.50	0.0000	1.48	up
242	TG (54:2-FA20:0)	1.40	0.0000	1.35	up
243	TG (54:2-FA20:1)	1.06	0.0016	1.26	up
244	TG (54:2-FA20:2)	1.24	0.0001	1.53	up
245	TG (54:3-FA16:0)	1.22	0.0001	1.20	up
246	TG (54:3-FA18:0)	1.07	0.0002	1.17	up
247	TG (54:3-FA18:3)	1.44	0.0000	1.72	up
248	TG (54:3-FA20:2)	1.07	0.0006	1.17	up
249	TG (54:3-FA20:3)	1.49	0.0000	1.62	up
250	TG (54:4-FA16:0)	1.37	0.0000	1.24	up
251	TG (54:4-FA18:0)	1.23	0.0001	1.23	up
252	TG (54:4-FA18:3)	1.37	0.0000	1.38	up
253	TG (54:4-FA20:1)	1.26	0.0004	1.20	up
254	TG (54:4-FA20:2)	1.01	0.0047	1.14	up
255	TG (54:4-FA20:3)	1.37	0.0000	1.29	up
256	TG (54:4-FA20:4)	1.36	0.0001	1.30	up
257	TG (54:4-FA22:4)	1.46	0.0000	1.51	up
258	TG (54:5-FA16:0)	1.52	0.0000	1.34	up
259	TG (54:5-FA18:0)	1.25	0.0000	1.34	up
260	TG (54:5-FA20:2)	1.34	0.0003	1.19	up
261	TG (54:5-FA20:3)	1.23	0.0003	1.20	up
262	TG (54:5-FA20:4)	1.36	0.0001	1.32	up

263	TG (54:5-FA20:5)	1.48	0.0000	1.49	up
264	TG (54:5-FA22:4)	1.26	0.0030	1.28	up
265	TG (54:5-FA22:5)	1.56	0.0000	1.73	up
266	TG (54:6-FA16:0)	1.31	0.0001	1.30	up
267	TG (54:6-FA16:1)	1.24	0.0064	1.17	up
268	TG (54:6-FA20:3)	1.27	0.0003	1.28	up
269	TG (54:6-FA20:4)	1.29	0.0006	1.24	up
270	TG (54:6-FA20:5)	1.50	0.0000	1.53	up
271	TG (54:6-FA22:5)	1.47	0.0000	1.40	up
272	TG (54:6-FA22:6)	1.42	0.0001	1.31	up
273	TG (54:7-FA20:4)	1.33	0.0008	1.24	up
274	TG (54:7-FA20:5)	1.50	0.0000	1.48	up
275	TG (54:7-FA22:5)	1.30	0.0011	1.28	up
276	TG (54:7-FA22:6)	1.45	0.0000	1.39	up
277	TG (54:8-FA20:5)	1.41	0.0000	1.48	up
278	TG (54:8-FA22:6)	1.27	0.0002	1.31	up
279	TG (55:1-FA16:0)	1.27	0.0059	1.24	up
280	TG (55:2-FA18:2)	1.15	0.0010	1.37	up
281	TG (55:7-FA22:6)	1.19	0.0010	1.33	up
282	TG (56:1-FA16:0)	1.29	0.0021	1.33	up
283	TG (56:1-FA18:1)	1.03	0.0118	1.25	up
284	TG (56:2-FA16:0)	1.28	0.0003	1.30	up
285	TG (56:3-FA16:0)	1.36	0.0001	1.28	up
286	TG (56:3-FA18:0)	1.21	0.0001	1.27	up
287	TG (56:3-FA20:2)	1.01	0.0004	1.23	up
288	TG (56:4-FA16:0)	1.22	0.0032	1.18	up
289	TG (56:4-FA18:0)	1.24	0.0001	1.26	up
290	TG (56:4-FA20:3)	1.25	0.0001	1.26	up
291	TG (56:4-FA20:4)	1.23	0.0001	1.27	up
292	TG (56:4-FA22:4)	1.41	0.0000	1.36	up
293	TG (56:5-FA16:0)	1.51	0.0000	1.38	up
294	TG (56:5-FA18:0)	1.44	0.0000	1.41	up
295	TG (56:5-FA18:1)	1.07	0.0004	1.24	up
296	TG (56:5-FA20:1)	1.15	0.0019	1.26	up
297	TG (56:5-FA20:4)	1.24	0.0000	1.30	up
298	TG (56:5-FA22:4)	1.26	0.0003	1.27	up
299	TG (56:5-FA22:5)	1.50	0.0000	1.91	up
300	TG (56:6-FA16:0)	1.44	0.0001	1.35	up
301	TG (56:6-FA18:0)	1.38	0.0000	1.25	up
302	TG (56:6-FA18:1)	1.32	0.0001	1.31	up
303	TG (56:6-FA18:2)	1.04	0.0004	1.27	up
304	TG (56:6-FA20:4)	1.01	0.0012	1.19	up
305	TG (56:6-FA20:5)	1.54	0.0000	1.51	up
306	TG (56:6-FA22:4)	1.15	0.0020	1.20	up

307	TG (56:6-FA22:5)	1.45	0.0000	1.39	up
308	TG (56:6-FA22:6)	1.53	0.0000	1.36	up
309	TG (56:7-FA16:0)	1.46	0.0000	1.34	up
310	TG (56:7-FA16:1)	1.05	0.0004	1.30	up
311	TG (56:7-FA18:0)	1.38	0.0000	1.37	up
312	TG (56:7-FA18:1)	1.50	0.0000	1.37	up
313	TG (56:7-FA18:2)	1.24	0.0008	1.19	up
314	TG (56:7-FA18:3)	1.06	0.0002	1.28	up
315	TG (56:7-FA20:5)	1.32	0.0000	1.32	up
316	TG (56:7-FA22:6)	1.55	0.0000	1.36	up
317	TG (56:8-FA16:0)	1.46	0.0000	1.32	up
318	TG (56:8-FA18:1)	1.16	0.0007	1.26	up
319	TG (56:8-FA18:2)	1.54	0.0000	1.40	up
320	TG (56:8-FA18:3)	1.24	0.0004	1.35	up
321	TG (56:8-FA20:4)	1.13	0.0017	1.25	up
322	TG (56:8-FA20:5)	1.12	0.0007	1.24	up
323	TG (56:8-FA22:5)	1.24	0.0006	1.24	up
324	TG (56:8-FA22:6)	1.39	0.0000	1.29	up
325	TG (56:9-FA20:5)	1.01	0.0026	1.25	up
326	TG (56:9-FA22:6)	1.62	0.0000	1.54	up
327	TG (57:3-FA18:2)	1.10	0.0178	1.24	up
328	TG (58:10-FA20:4)	1.34	0.0000	1.38	up
329	TG (58:10-FA20:5)	1.25	0.0000	1.57	up
330	TG (58:10-FA22:6)	1.42	0.0000	1.43	up
331	TG (58:5-FA18:1)	1.02	0.0129	1.14	up
332	TG (58:6-FA16:0)	1.41	0.0001	1.37	up
333	TG (58:6-FA18:0)	1.53	0.0000	1.38	up
334	TG (58:6-FA18:1)	1.43	0.0000	1.31	up
335	TG (58:6-FA22:5)	1.49	0.0000	1.46	up
336	TG (58:7-FA16:0)	1.45	0.0001	1.34	up
337	TG (58:7-FA18:0)	1.47	0.0000	1.37	up
338	TG (58:7-FA18:1)	1.02	0.0049	1.19	up
339	TG (58:7-FA18:2)	1.38	0.0001	1.24	up
340	TG (58:7-FA22:5)	1.29	0.0000	1.32	up
341	TG (58:8-FA18:2)	1.25	0.0000	1.34	up
342	TG (58:8-FA20:4)	1.26	0.0000	1.30	up
343	TG (58:8-FA22:5)	1.07	0.0029	1.19	up
344	TG (58:8-FA22:6)	1.08	0.0001	1.29	up
345	TG (58:9-FA20:4)	1.13	0.0010	1.20	up
346	TG (60:10-FA22:5)	1.12	0.0031	1.25	up
347	TG (60:10-FA22:6)	1.25	0.0000	1.50	up
348	TG (60:11-FA22:5)	1.52	0.0000	1.51	up
349	TG (60:11-FA22:6)	1.50	0.0000	1.45	up

Supplementary Table2 Lipid metabolites in the association between dietary nutrients and CMM risk

Factors	Metabolite	Cor	P (cor)	P (mediate)	P (total effect)	Prop.Mediated
Fatty acid	DG (16:0/18:3)	-0.13	0.01	0	0	0.28
Fatty acid	HexCer (d18:1/24:1)	0.13	0.01	0.01	0	0.13
Fatty acid	LysoPC (19:0)	0.12	0.01	0	0	0.16
Fatty acid	LysoPC (22:0)	0.09	0.04	0.04	0	0.18
Fatty acid	LysoPC (24:1)	0.09	0.04	0.04	0	0.21
Fatty acid	LysoPC (O-22:0)	0.11	0.02	0.04	0.01	0.11
Fatty acid	LysoPE (20:2)	0.14	0.00	0	0	0.18
Fatty acid	PC (18:1/18:2)	0.12	0.01	0.02	0	0.23
Fatty acid	PC (18:1/20:2)	0.13	0.00	0.04	0.03	0.11
Fatty acid	PC (18:1/20:4)	0.11	0.02	0.04	0	0.15
Fatty acid	PC (18:1/22:4)	0.16	0.00	0	0	0.19
Fatty acid	PC (18:1/22:5)	0.10	0.03	0.04	0	0.16
Fatty acid	PC (18:1/22:6)	0.10	0.03	0.03	0	0.15
Fatty acid	PC (18:2/20:2)	0.10	0.03	0.03	0	0.18
Fatty acid	PC (18:2/22:4)	0.11	0.02	0.03	0	0.12
Fatty acid	PC (18:2/22:6)	0.10	0.03	0.02	0	0.13
Fatty acid	PC (20:0/20:3)	0.11	0.02	0.03	0	0.12
Fatty acid	PC (O-18:1/18:2)	0.12	0.01	0	0	0.21
Fatty acid	PC (P-16:0/18:2)	0.10	0.03	0.05	0	0.12
Fatty acid	PC (P-16:0/20:2)	0.12	0.01	0.02	0	0.14
Fatty acid	PC (P-18:0/18:2)	0.10	0.03	0.02	0	0.12
Fatty acid	PC (P-18:1/18:1)	0.13	0.01	0	0	0.25
Fatty acid	PE (18:0/22:6)	-0.11	0.02	0.01	0	0.20
Fatty acid	PI (14:0/18:0)	-0.11	0.02	0.01	0	0.19
Fatty acid	PI (18:1/18:1)	0.15	0.00	0.01	0	0.19
Fatty acid	PI (18:1/18:2)	0.15	0.00	0	0	0.32
Fatty acid	PI (18:1/20:3)	0.15	0.00	0	0	0.27
Fatty acid	PI (18:2/18:2)	0.12	0.01	0.01	0	0.23
Fatty acid	PS (20:0/18:1)	0.13	0.00	0.01	0	0.19
Fatty acid	TG (52:6-FA16:0)	-0.11	0.02	0.01	0	0.17
Fatty acid	TG (52:6-FA18:3)	-0.10	0.03	0.01	0	0.14
Fatty acid	TG (54:4-FA18:3)	-0.09	0.04	0.03	0	0.17
Fatty acid	TG (54:6-FA16:0)	-0.10	0.04	0.01	0	0.14
Fatty acid	TG (54:8-FA20:5)	-0.09	0.04	0.01	0	0.17
Fatty acid	TG (56:7-FA18:3)	-0.09	0.04	0.03	0	0.14
Fatty acid	TG (56:9-FA20:5)	-0.10	0.04	0.01	0	0.11
Fatty acid	TG (56:9-FA22:6)	-0.11	0.01	0	0	0.25
MUFA	DG (16:0/18:3)	-0.12	0.01	0.00	0.00	0.27
MUFA	HexCer (d18:1/24:1)	0.12	0.01	0.02	0.00	0.13
MUFA	LysoPC (19:0)	0.12	0.01	0.00	0.00	0.16
MUFA	LysoPC (22:0)	0.09	0.04	0.04	0.00	0.17
MUFA	LysoPC (24:1)	0.10	0.04	0.04	0.00	0.22

MUFA	LysoPC (O-22:0)	0.11	0.02	0.04	0.01	0.10
MUFA	LysoPE (20:2)	0.13	0.00	0.01	0.00	0.18
MUFA	PC (18:1/18:2)	0.12	0.01	0.03	0.00	0.23
MUFA	PC (18:1/20:2)	0.14	0.00	0.04	0.03	0.11
MUFA	PC (18:1/20:4)	0.11	0.02	0.04	0.00	0.15
MUFA	PC (18:1/22:4)	0.16	0.00	0.00	0.00	0.19
MUFA	PC (18:1/22:6)	0.09	0.05	0.04	0.00	0.14
MUFA	PC (18:2/18:2)	0.09	0.05	0.03	0.00	0.13
MUFA	PC (18:2/20:2)	0.11	0.02	0.02	0.00	0.20
MUFA	PC (18:2/22:4)	0.10	0.03	0.02	0.00	0.11
MUFA	PC (18:2/22:6)	0.10	0.04	0.02	0.00	0.13
MUFA	PC (20:0/18:1)	0.11	0.01	0.03	0.04	0.09
MUFA	PC (20:0/20:3)	0.10	0.03	0.05	0.00	0.12
MUFA	PC (O-18:1/18:2)	0.14	0.00	0.00	0.00	0.24
MUFA	PC (P-16:0/18:2)	0.11	0.02	0.02	0.00	0.14
MUFA	PC (P-16:0/20:2)	0.13	0.01	0.01	0.00	0.15
MUFA	PC (P-18:0/18:2)	0.11	0.02	0.02	0.00	0.13
MUFA	PC (P-18:1/18:1)	0.14	0.00	0.00	0.00	0.28
MUFA	PE (18:0/22:6)	-0.11	0.02	0.01	0.00	0.19
MUFA	PI (14:0/18:0)	-0.11	0.02	0.02	0.00	0.19
MUFA	PI (18:1/18:1)	0.16	0.00	0.00	0.00	0.20
MUFA	PI (18:1/20:3)	0.14	0.00	0.01	0.00	0.25
MUFA	P I(18:1/20:4)	0.14	0.00	0.00	0.00	0.29
MUFA	PS (20:0/18:1)	0.13	0.01	0.02	0.00	0.18
MUFA	TG (52:6-FA16:0)	-0.10	0.04	0.01	0.00	0.16
MUFA	TG (56:9-FA20:5)	-0.10	0.04	0.01	0.00	0.11
MUFA	TG (56:9-FA22:6)	-0.10	0.03	0.01	0.00	0.23
PUFA	LysoPC (24:0)	0.10	0.02	0.03	0.00	0.19
PUFA	LysoPC (O-22:0)	0.12	0.01	0.02	0.01	0.13
PUFA	LysoPG (18:0)	-0.10	0.03	0.01	0.00	0.19
PUFA	PC (18:1/20:2)	0.14	0.00	0.03	0.03	0.12
PUFA	PC (18:1/20:4)	0.11	0.02	0.02	0.00	0.16
PUFA	PC (18:1/22:5)	0.11	0.02	0.01	0.00	0.19
PUFA	PC (18:1/22:6)	0.11	0.02	0.03	0.00	0.18
PUFA	PC (20:0/20:3)	0.12	0.01	0.02	0.00	0.15
PUFA	PC (O-18:1/18:2)	0.10	0.02	0.01	0.00	0.20
PUFA	PE (18:0/22:6)	-0.12	0.01	0.00	0.00	0.24
PUFA	PI (18:1/18:1)	0.14	0.00	0.02	0.00	0.19
PUFA	PS (20:0/18:1)	0.13	0.01	0.01	0.00	0.20
PUFA	TG (52:4-FA18:3)	-0.09	0.05	0.02	0.00	0.16
PUFA	TG (52:5-FA16:0)	-0.10	0.04	0.01	0.00	0.17
PUFA	TG (52:5-FA18:3)	-0.10	0.04	0.02	0.00	0.15
PUFA	TG (52:6-FA18:3)	-0.11	0.02	0.00	0.00	0.17
PUFA	TG (54:3-FA18:3)	-0.09	0.04	0.01	0.00	0.22

PUFA	TG (54:4-FA18:3)	-0.11	0.02	0.00	0.00	0.22
PUFA	TG (54:4-FA20:1)	-0.09	0.04	0.01	0.00	0.14
PUFA	TG (54:6-FA16:0)	-0.11	0.02	0.00	0.00	0.17
PUFA	TG (54:8-FA20:5)	-0.11	0.02	0.00	0.00	0.21
PUFA	TG (56:6-FA22:6)	-0.10	0.04	0.03	0.00	0.23
PUFA	TG (56:7-FA18:3)	-0.10	0.03	0.02	0.00	0.17
PUFA	TG (56:7-FA22:6)	-0.10	0.04	0.02	0.00	0.23
PUFA	TG (56:8-FA16:0)	-0.09	0.04	0.03	0.00	0.18
PUFA	TG (56:8-FA18:2)	-0.10	0.02	0.00	0.00	0.13
PUFA	TG (58:8-FA22:6)	-0.10	0.04	0.01	0.00	0.19
PUFA	TG (60:11-FA22:6)	-0.10	0.03	0.01	0.00	0.24
VE	CE (20:5)	-0.10	0.04	0.02	0.00	0.11
VE	Cer (d18:0/22:1)	-0.11	0.02	0.01	0.00	0.18
VE	DG (16:0/18:1)	-0.14	0.00	0.00	0.00	0.24
VE	DG (16:0/18:3)	-0.12	0.01	0.00	0.00	0.23
VE	DG (18:0/18:1)	-0.11	0.02	0.01	0.00	0.17
VE	HexCer (d18:1/24:1)	0.13	0.01	0.05	0.01	0.11
VE	LysoPC (19:0)	0.10	0.03	0.04	0.00	0.12
VE	LysoPC (20:0)	0.10	0.03	0.02	0.00	0.13
VE	LysoPC (20:5)	-0.11	0.02	0.01	0.00	0.18
VE	LysoPC (22:0)	0.14	0.00	0.00	0.00	0.22
VE	LysoPC (24:0)	0.12	0.01	0.02	0.00	0.17
VE	LysoPC (24:1)	0.11	0.02	0.04	0.00	0.21
VE	LysoPC (O-22:0)	0.16	0.00	0.02	0.02	0.12
VE	LysoPE (20:2)	0.14	0.00	0.01	0.00	0.15
VE	LysoPE (20:5)	-0.10	0.04	0.04	0.00	0.09
VE	LysoPE (22:0)	0.11	0.02	0.03	0.00	0.12
VE	LysoPG (18:0)	-0.11	0.01	0.00	0.00	0.17
VE	PC (16:0/20:5)	-0.11	0.02	0.00	0.00	0.16
VE	PC (18:0/20:5)	-0.09	0.04	0.02	0.00	0.09
VE	PC (18:1/18:2)	0.13	0.00	0.03	0.00	0.21
VE	PC (18:1/20:4)	0.11	0.02	0.02	0.00	0.12
VE	PC (18:1/22:4)	0.13	0.01	0.01	0.00	0.13
VE	PC (18:1/22:6)	0.13	0.00	0.01	0.00	0.16
VE	PC (18:2/22:4)	0.12	0.01	0.03	0.00	0.11
VE	PC (18:2/22:6)	0.11	0.02	0.02	0.00	0.12
VE	PC (20:0/20:3)	0.11	0.02	0.03	0.00	0.10
VE	PC (P-18:1/18:1)	0.10	0.03	0.04	0.00	0.16
VE	PE (16:0/16:0)	-0.11	0.02	0.01	0.00	0.16
VE	PE (16:0/20:5)	-0.12	0.01	0.00	0.02	0.10
VE	PE (18:0/20:5)	-0.12	0.01	0.00	0.00	0.14
VE	PE (18:0/22:5)	-0.11	0.01	0.01	0.01	0.11
VE	PE (18:0/22:6)	-0.13	0.00	0.00	0.00	0.20
VE	PG (18:0/18:1)	-0.11	0.02	0.01	0.00	0.11

VE	PG (18:0/18:2)	-0.10	0.02	0.01	0.00	0.11
VE	PI (18:1/18:1)	0.11	0.02	0.03	0.00	0.12
VE	PI (18:1/18:2)	0.15	0.00	0.00	0.00	0.28
VE	PI (18:1/20:3)	0.17	0.00	0.00	0.00	0.26
VE	PI (18:1/20:4)	0.17	0.00	0.00	0.00	0.29
VE	PI (18:2/18:2)	0.13	0.00	0.01	0.00	0.21
VE	PS (20:0/18:1)	0.10	0.03	0.04	0.00	0.12
VE	TG (46:3-FA18:3)	-0.09	0.05	0.02	0.00	0.10
VE	TG (48:0-FA16:0)	-0.10	0.02	0.01	0.00	0.15
VE	TG (48:2-FA14:0)	-0.09	0.05	0.03	0.00	0.10
VE	TG (48:2-FA18:2)	-0.10	0.03	0.02	0.00	0.12
VE	TG (48:3-FA14:0)	-0.11	0.02	0.01	0.00	0.12
VE	TG (48:3-FA16:0)	-0.10	0.03	0.01	0.00	0.11
VE	TG (48:3-FA18:3)	-0.11	0.02	0.00	0.00	0.13
VE	TG (48:4-FA14:0)	-0.09	0.04	0.02	0.02	0.07
VE	TG (49:3-FA16:0)	-0.09	0.04	0.02	0.03	0.07
VE	TG (50:0-FA14:0)	-0.09	0.05	0.01	0.00	0.12
VE	TG (50:0-FA16:0)	-0.11	0.02	0.01	0.00	0.16
VE	TG (50:0-FA18:0)	-0.11	0.02	0.00	0.00	0.17
VE	TG (50:1-FA14:0)	-0.09	0.05	0.04	0.00	0.12
VE	TG (50:1-FA16:0)	-0.12	0.01	0.01	0.00	0.17
VE	TG (50:1-FA16:1)	-0.09	0.04	0.02	0.00	0.12
VE	TG (50:1-FA18:0)	-0.09	0.05	0.03	0.00	0.12
VE	TG (50:1-FA18:1)	-0.12	0.01	0.01	0.00	0.17
VE	TG (50:2-FA16:0)	-0.11	0.01	0.00	0.00	0.15
VE	TG (50:2-FA18:0)	-0.10	0.03	0.03	0.00	0.13
VE	TG (50:2-FA18:2)	-0.13	0.01	0.00	0.00	0.21
VE	TG (50:3-FA16:0)	-0.13	0.01	0.00	0.00	0.17
VE	TG (50:3-FA18:0)	-0.11	0.02	0.00	0.00	0.15
VE	TG (50:3-FA18:2)	-0.10	0.03	0.05	0.01	0.09
VE	TG (50:3-FA18:3)	-0.14	0.00	0.00	0.00	0.21
VE	TG (50:4-FA16:0)	-0.12	0.01	0.00	0.01	0.11
VE	TG (50:4-FA16:1)	-0.11	0.01	0.03	0.02	0.09
VE	TG (50:4-FA18:1)	-0.10	0.04	0.02	0.01	0.09
VE	TG (50:4-FA18:3)	-0.11	0.02	0.00	0.00	0.11
VE	TG (50:5-FA14:0)	-0.09	0.04	0.02	0.00	0.10
VE	TG (50:5-FA18:2)	-0.09	0.05	0.04	0.01	0.09
VE	TG (50:5-FA18:3)	-0.10	0.04	0.03	0.02	0.08
VE	TG (50:5-FA20:5)	-0.12	0.01	0.01	0.00	0.17
VE	TG (52:0-FA16:0)	-0.10	0.03	0.00	0.00	0.17
VE	TG (52:0-FA18:0)	-0.10	0.03	0.01	0.00	0.17
VE	TG (52:0-FA20:0)	-0.11	0.02	0.01	0.00	0.17
VE	TG (52:1-FA16:0)	-0.11	0.02	0.01	0.00	0.17
VE	TG (52:1-FA16:1)	-0.09	0.04	0.03	0.00	0.12

VE	TG (52:1-FA18:0)	-0.11	0.02	0.02	0.00	0.18
VE	TG (52:1-FA18:1)	-0.11	0.02	0.00	0.00	0.18
VE	TG (52:1-FA20:1)	-0.11	0.02	0.02	0.00	0.16
VE	TG (52:2-FA16:0)	-0.11	0.02	0.02	0.00	0.13
VE	TG (52:2-FA16:1)	-0.10	0.03	0.03	0.00	0.11
VE	TG (52:2-FA18:0)	-0.12	0.01	0.00	0.00	0.21
VE	TG (52:2-FA18:1)	-0.11	0.02	0.02	0.00	0.12
VE	TG (52:2-FA18:2)	-0.12	0.01	0.00	0.00	0.25
VE	TG (52:2-FA20:0)	-0.10	0.02	0.04	0.00	0.11
VE	TG (52:2-FA20:2)	-0.10	0.02	0.02	0.00	0.16
VE	TG (52:3-FA16:0)	-0.11	0.02	0.01	0.00	0.12
VE	TG (52:3-FA18:0)	-0.13	0.00	0.00	0.00	0.21
VE	TG (52:3-FA18:1)	-0.10	0.03	0.03	0.01	0.09
VE	TG (52:3-FA18:2)	-0.11	0.02	0.01	0.00	0.11
VE	TG (52:3-FA18:3)	-0.13	0.00	0.00	0.00	0.22
VE	TG (52:3-FA20:0)	-0.10	0.03	0.02	0.00	0.11
VE	TG (52:3-FA20:1)	-0.10	0.04	0.03	0.01	0.08
VE	TG (52:3-FA20:3)	-0.11	0.02	0.01	0.00	0.17
VE	TG (52:4-FA16:0)	-0.12	0.01	0.00	0.00	0.15
VE	TG (52:4-FA18:0)	-0.13	0.00	0.00	0.00	0.16
VE	TG (52:4-FA18:1)	-0.13	0.00	0.00	0.00	0.14
VE	TG (52:4-FA18:3)	-0.13	0.00	0.00	0.00	0.17
VE	TG (52:4-FA20:0)	-0.11	0.02	0.01	0.00	0.14
VE	TG (52:4-FA20:4)	-0.10	0.03	0.01	0.00	0.14
VE	TG (52:4-FA22:4)	-0.10	0.02	0.01	0.00	0.13
VE	TG (52:5-FA16:0)	-0.13	0.01	0.00	0.00	0.17
VE	TG (52:5-FA18:2)	-0.12	0.01	0.01	0.00	0.13
VE	TG (52:5-FA18:3)	-0.13	0.01	0.00	0.00	0.15
VE	TG (52:5-FA20:5)	-0.11	0.01	0.01	0.00	0.13
VE	TG (52:5-FA22:5)	-0.13	0.00	0.00	0.00	0.19
VE	TG (52:6-FA16:0)	-0.12	0.01	0.00	0.00	0.16
VE	TG (52:6-FA18:3)	-0.12	0.01	0.00	0.00	0.14
VE	TG (52:6-FA20:5)	-0.11	0.02	0.02	0.00	0.13
VE	TG (52:7-FA16:0)	-0.11	0.02	0.00	0.01	0.11
VE	TG (52:7-FA20:5)	-0.12	0.01	0.01	0.00	0.13
VE	TG (52:7-FA22:6)	-0.10	0.04	0.02	0.00	0.09
VE	TG (54:0-FA16:0)	-0.12	0.01	0.00	0.00	0.18
VE	TG (54:1-FA16:0)	-0.10	0.03	0.02	0.00	0.16
VE	TG (54:1-FA18:1)	-0.10	0.04	0.01	0.00	0.15
VE	TG (54:1-FA20:0)	-0.10	0.03	0.05	0.00	0.14
VE	TG (54:2-FA16:0)	-0.10	0.02	0.04	0.00	0.13
VE	TG (54:2-FA18:0)	-0.09	0.04	0.04	0.00	0.12
VE	TG (54:2-FA18:2)	-0.12	0.01	0.01	0.00	0.22
VE	TG (54:2-FA20:0)	-0.10	0.04	0.03	0.00	0.15

VE	TG(54:2-FA20:2)	-0.11	0.02	0.01	0.00	0.21
VE	TG (54:3-FA16:0)	-0.10	0.03	0.05	0.00	0.13
VE	TG (54:3-FA18:3)	-0.12	0.01	0.00	0.00	0.22
VE	TG (54:3-FA20:3)	-0.12	0.01	0.00	0.00	0.23
VE	TG (54:4-FA16:0)	-0.11	0.02	0.03	0.00	0.14
VE	TG (54:4-FA18:0)	-0.09	0.05	0.03	0.00	0.12
VE	TG (54:4-FA18:3)	-0.12	0.01	0.00	0.00	0.18
VE	TG (54:4-FA20:1)	-0.13	0.00	0.00	0.00	0.14
VE	TG (54:4-FA20:3)	-0.11	0.02	0.04	0.00	0.15
VE	TG (54:4-FA22:4)	-0.11	0.02	0.02	0.00	0.17
VE	TG(54:5-FA16:0)	-0.11	0.01	0.02	0.00	0.17
VE	TG (54:5-FA18:0)	-0.09	0.04	0.02	0.00	0.14
VE	TG (54:5-FA20:2)	-0.11	0.02	0.01	0.00	0.12
VE	TG (54:5-FA20:3)	-0.10	0.03	0.02	0.00	0.12
VE	TG (54:5-FA20:5)	-0.13	0.01	0.01	0.00	0.21
VE	TG (54:5-FA22:5)	-0.14	0.00	0.00	0.00	0.23
VE	TG (54:6-FA16:0)	-0.11	0.02	0.01	0.00	0.13
VE	TG (54:6-FA20:3)	-0.10	0.03	0.02	0.00	0.11
VE	TG (54:6-FA20:5)	-0.13	0.01	0.00	0.00	0.21
VE	TG (54:6-FA22:5)	-0.12	0.01	0.01	0.00	0.16
VE	TG (54:6-FA22:6)	-0.11	0.02	0.01	0.00	0.15
VE	TG (54:7-FA20:4)	-0.11	0.02	0.01	0.00	0.12
VE	TG (54:7-FA20:5)	-0.14	0.00	0.00	0.00	0.23
VE	TG (54:7-FA22:5)	-0.11	0.01	0.01	0.00	0.12
VE	TG (54:7-FA22:6)	-0.12	0.01	0.01	0.00	0.16
VE	TG (54:8-FA20:5)	-0.15	0.00	0.00	0.00	0.21
VE	TG (54:8-FA22:6)	-0.12	0.01	0.00	0.00	0.15
VE	TG (55:7-FA22:6)	-0.11	0.02	0.00	0.00	0.12
VE	TG (56:1-FA16:0)	-0.10	0.03	0.03	0.00	0.12
VE	TG (56:3-FA18:0)	-0.11	0.02	0.02	0.00	0.16
VE	TG (56:3-FA20:2)	-0.09	0.04	0.04	0.00	0.13
VE	TG (56:4-FA18:0)	-0.10	0.04	0.02	0.00	0.12
VE	TG (56:4-FA20:3)	-0.10	0.02	0.02	0.00	0.14
VE	TG (56:4-FA22:4)	-0.10	0.03	0.04	0.00	0.15
VE	TG (56:5-FA16:0)	-0.11	0.02	0.04	0.00	0.17
VE	TG (56:5-FA18:0)	-0.11	0.02	0.02	0.00	0.17
VE	TG (56:5-FA20:1)	-0.11	0.02	0.01	0.00	0.11
VE	TG (56:5-FA22:5)	-0.13	0.00	0.00	0.00	0.26
VE	TG (56:6-FA16:0)	-0.12	0.01	0.01	0.00	0.16
VE	TG (56:6-FA18:0)	-0.10	0.03	0.02	0.00	0.14
VE	TG (56:6-FA18:1)	-0.10	0.03	0.04	0.00	0.13
VE	TG (56:6-FA20:5)	-0.13	0.00	0.00	0.00	0.25
VE	TG (56:6-FA22:5)	-0.13	0.01	0.01	0.00	0.18
VE	TG (56:6-FA22:6)	-0.13	0.01	0.00	0.00	0.23

VE	TG (56:7-FA16:0)	-0.12	0.01	0.00	0.00	0.20
VE	TG (56:7-FA16:1)	-0.10	0.03	0.01	0.00	0.13
VE	TG (56:7-FA18:0)	-0.10	0.02	0.01	0.00	0.18
VE	TG (56:7-FA18:1)	-0.12	0.01	0.00	0.00	0.20
VE	TG (56:7-FA20:5)	-0.13	0.01	0.00	0.00	0.18
VE	TG (56:7-FA22:6)	-0.13	0.01	0.00	0.00	0.23
VE	TG (56:8-FA16:0)	-0.12	0.01	0.01	0.00	0.18
VE	TG (56:8-FA18:1)	-0.11	0.02	0.01	0.00	0.12
VE	TG (56:8-FA18:2)	-0.14	0.00	0.00	0.00	0.22
VE	TG (56:8-FA18:3)	-0.12	0.01	0.00	0.00	0.14
VE	TG (56:8-FA20:5)	-0.12	0.01	0.00	0.00	0.13
VE	TG (56:8-FA22:6)	-0.11	0.02	0.01	0.00	0.16
VE	TG (56:9-FA20:5)	-0.11	0.02	0.00	0.01	0.10
VE	TG (56:9-FA22:6)	-0.15	0.00	0.00	0.00	0.29
VE	TG (58:10-FA20:4)	-0.11	0.02	0.00	0.00	0.18
VE	TG (58:10-FA20:5)	-0.12	0.01	0.00	0.00	0.20
VE	TG (58:10-FA22:6)	-0.11	0.02	0.01	0.00	0.20
VE	TG (58:6-FA16:0)	-0.11	0.01	0.00	0.00	0.14
VE	TG (58:6-FA18:0)	-0.12	0.01	0.01	0.00	0.20
VE	TG (58:6-FA18:1)	-0.11	0.02	0.01	0.00	0.15
VE	TG (58:6-FA22:5)	-0.13	0.00	0.00	0.00	0.22
VE	TG (58:7-FA16:0)	-0.11	0.01	0.01	0.00	0.14
VE	TG (58:7-FA18:0)	-0.12	0.01	0.01	0.00	0.19
VE	TG (58:7-FA18:1)	-0.09	0.04	0.02	0.01	0.08
VE	TG (58:7-FA18:2)	-0.11	0.02	0.02	0.00	0.14
VE	TG (58:7-FA22:5)	-0.13	0.01	0.00	0.00	0.18
VE	TG (58:8-FA22:5)	-0.10	0.04	0.02	0.01	0.09
VE	TG (58:8-FA22:6)	-0.09	0.05	0.01	0.00	0.14
VE	TG (60:11-FA22:5)	-0.14	0.00	0.00	0.00	0.23
VE	TG (60:11-FA22:6)	-0.13	0.00	0.00	0.00	0.24
